# Intrinsic Epigenetic Regulation of the D4Z4 Macrosatellite Repeat in a Transgenic Mouse Model for FSHD

**DOI:** 10.1371/journal.pgen.1003415

**Published:** 2013-04-04

**Authors:** Yvonne D. Krom, Peter E. Thijssen, Janet M. Young, Bianca den Hamer, Judit Balog, Zizhen Yao, Lisa Maves, Lauren Snider, Paul Knopp, Peter S. Zammit, Tonnie Rijkers, Baziel G. M. van Engelen, George W. Padberg, Rune R. Frants, Rabi Tawil, Stephen J. Tapscott, Silvère M. van der Maarel

**Affiliations:** 1 Department of Human Genetics, Leiden University Medical Center, Leiden, The Netherlands; 2 Division of Human Biology, Fred Hutchinson Cancer Research Center, Seattle, Washington, United States of America; 3 King's College London, Randall Division of Cell and Molecular Biophysics, Guy's Campus, London, United Kingdom; 4 Neuromuscular Centre Nijmegen, Department of Neurology, Radboud University Nijmegen Medical Centre, Nijmegen, The Netherlands; 5 Neuromuscular Disease Unit, Department of Neurology, University of Rochester Medical Center, Rochester, New York, United States of America; The Hospital for Sick Children and University of Toronto, Canada

## Abstract

Facioscapulohumeral dystrophy (FSHD) is a progressive muscular dystrophy caused by decreased epigenetic repression of the D4Z4 macrosatellite repeats and ectopic expression of *DUX4*, a retrogene encoding a germline transcription factor encoded in each repeat. Unaffected individuals generally have more than 10 repeats arrayed in the subtelomeric region of chromosome 4, whereas the most common form of FSHD (FSHD1) is caused by a contraction of the array to fewer than 10 repeats, associated with decreased epigenetic repression and variegated expression of DUX4 in skeletal muscle. We have generated transgenic mice carrying D4Z4 arrays from an FSHD1 allele and from a control allele. These mice recapitulate important epigenetic and DUX4 expression attributes seen in patients and controls, respectively, including high DUX4 expression levels in the germline, (incomplete) epigenetic repression in somatic tissue, and FSHD–specific variegated DUX4 expression in sporadic muscle nuclei associated with D4Z4 chromatin relaxation. In addition we show that DUX4 is able to activate similar functional gene groups in mouse muscle cells as it does in human muscle cells. These transgenic mice therefore represent a valuable animal model for FSHD and will be a useful resource to study the molecular mechanisms underlying FSHD and to test new therapeutic intervention strategies.

## Author Summary

Facioscapulohumeral dystrophy (FSHD) is a progressive muscle disorder that is associated with contraction and chromatin relaxation of the D4Z4 macrosatellite repeat on chromosome 4q. Each unit of the repeat contains a copy of the primate-specific *DUX4* retrogene, encoding a germline transcription factor that is repressed in somatic tissue. In FSHD, somatic repression of the *DUX4* gene is compromised, leading to a variegated expression pattern of DUX4 in muscle cells. The complex (epi)genetic etiology of FSHD has long hampered the generation of a faithful animal model, and thus far the role of FSHD candidate genes has only been studied in model organisms by overexpression approaches. Here we present two transgenic mouse models containing either patient- or control-sized D4Z4 repeats. In our mice, the regulation of the FSHD locus is preserved in both lines, and only in the disease model somatic derepression and variegated expression of DUX4 is observed. These mice thus reflect many aspects of the complex regulation of DUX4 expression in humans. These models may therefore become valuable tools in understanding the *in vivo* regulation and function of DUX4, its role in FSHD, and the evaluation of therapeutic strategies.

## Introduction

Each unit of the D4Z4 macrosatellite repeat contains a copy of the *DUX4* retrogene that encodes a double homeobox transcription factor [1]–[4]. DUX4 is highly expressed in the germline and epigenetically repressed in most somatic tissues, including skeletal muscle [5], [6]. Recently, we and others showed that facioscapulohumeral dystrophy (FSHD), a muscular dystrophy predominantly affecting facial and upper extremity muscles [7], is caused by D4Z4 repeat contraction-dependent (FSHD1) or –independent (FSHD2) chromatin relaxation in somatic tissues and low levels of *DUX4* mRNA expression in skeletal muscle [5], [8]–[10]. On normal chromosomes 4, the D4Z4 repeat array varies between 11–100 units, while in FSHD1 one of the chromosomes 4 has an array of 1–10 units associated with a less repressive D4Z4 chromatin structure [11]–[13]. In FSHD2, the D4Z4 repeats are not contracted and D4Z4 chromatin relaxation can be observed on all arrays [8]. The low abundance of *DUX4* mRNA in FSHD muscle tissue represents a variegated pattern of expression with abundant DUX4 protein expressed in a small number of nuclei [6], [14], presumably due to an occasional escape from the inefficient epigenetic repression. The polyadenylation (pA) site for *DUX4* mRNA is in the DNA sequence immediately telomeric to the last D4Z4 repeat unit and chromosome 4 haplotypes non-permissive for FSHD contain inactivating polymorphisms at the pA site, explaining the haplotype-specificity of this disease [2], [15], [16].

When expressed in skeletal muscle, the DUX4 transcription factor activates genes normally expressed in the germline, essentially inducing a stem cell program in the postmitotic muscle cell. In addition, DUX4 binds and transcriptionally activates endogenous retrotransposons and simultaneously blocks the innate immune response, at least in part through the transcriptional activation of a beta-defensin [5].

The parental gene to primate *DUX4* was necessarily expressed in the germline, since germline retro-transposition was necessary for it to enter the primate lineage. As a retrogene, however, *DUX4* was dissociated from its evolved enhancers, promoters, and pA site, suggesting that the *DUX4* retrogene adopted independently evolved mechanisms to regulate its developmental expression [17]. One hypothesis is that the repression of DUX4 transcription in most somatic tissues relies on an independently evolved mechanism of repeat-mediated silencing. If true, then it is to be expected that somatic *DUX4* silencing is an evolutionary conserved mechanism that can be recapitulated in other species such as mouse.

No studies have yet addressed whether the D4Z4 repeat array and its flanking sequence is sufficient to accurately reproduce the developmental pattern of DUX4 expression, nor whether the FSHD mutation can recapitulate the decreased epigenetic repression and variegated DUX4 expression in a mouse model. The latter question is particularly relevant as both primates and rodents have lost the parental copy of the *DUX4* retrogene [18], but only primates have DUX4 integrated in the context of a D4Z4 macrosatellite repeat array and it is not known whether integration of the human array in mice can – at least in part – recapitulate the molecular characteristics of FSHD.

Here we report the generation and molecular characterization of two transgenic mouse lines: one carrying a D4Z4 genomic region from a contracted pathogenic FSHD1 allele and one carrying a normal sized, non-pathogenic allele. Our data suggest that somatic epigenetic silencing of *DUX4* indeed is an evolutionary conserved mechanism and that contracted D4Z4 repeat arrays are silenced less efficiently, leading to a variegated expression pattern of DUX4 protein in skeletal muscle nuclei.

## Results

### Generation of transgenic mouse models

**Figure 1:**
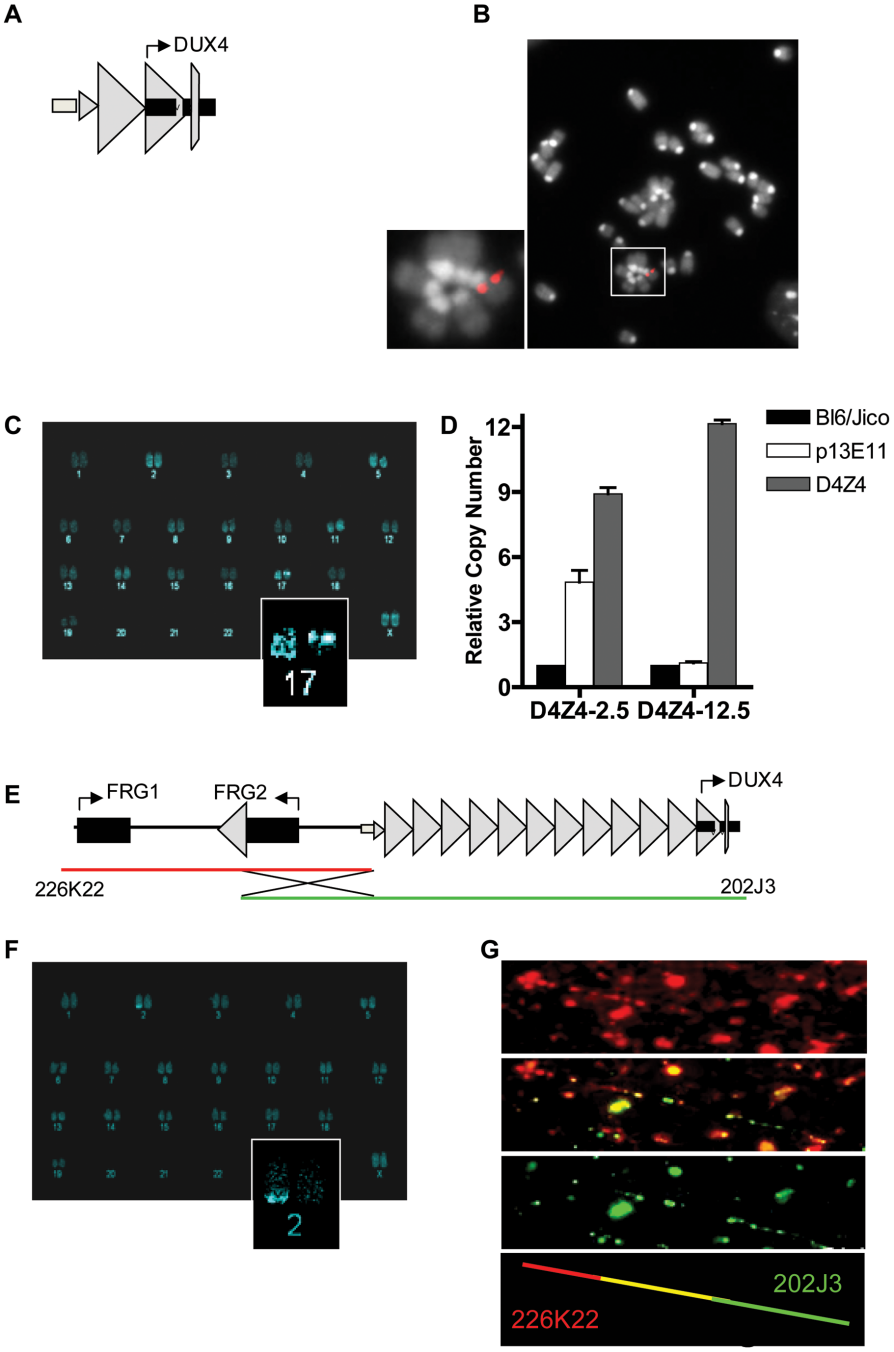
Integration site and copy number of D4Z4-2.5 and D4Z4-12.5 constructs in the mouse genome. A) Schematic draw of the L42 *Eco*RI fragment used to generate the D4Z4-2.5 mouse line B) Metaphase spread of D4Z4-2.5 fibroblasts co-stained with dapi and the CY3 labeled L42 probe shows integration at a single pair of chromosomes C) COBRA-FISH analysis on D4Z4-2.5 fibroblast metaphase spreads probed with biotinylated-L42 fragments shows integration of L42 on chr17; D) Detection of copy number of the integrated fragments in both mouse models by MLPA analysis. The probe mix contained three probes specific for wild type alleles, one probe designed against the human p13E-11 region and one probe against D4Z4 E) Schematic draw of PAC clones used to generate the D4Z4-12.5 mouse; F) COBRA-FISH analysis on D4Z4-12.5 fibroblast metaphase spreads probed with a biotinylated PAC clone shows integration of the PAC clone on chr2; G) Fiber-FISH analysis of D4Z4-12.5 fibroblasts. Both PAC clones, labeled and hybridized to DNA fibers, were shown to be recombined during integration into the mouse genome.

To determine whether the D4Z4 repeat with the *DUX4* retrogene contains the regulatory elements necessary for germline expression and copy-number dependent somatic epigenetic repression, we generated two transgenic mouse lines. One line carries an *Eco*RI fragment derived from the lambda-42 (L42) clone of an FSHD1 allele (Figure 1A). This allele is of the FSHD-permissive 4A161 background, containing the *DUX4* pA signal, but lacking the more downstream exons 6 and 7 [6], and contains two-and-a-half copies of the D4Z4 unit and flanking sequences (herein referred to as D4Z4-2.5 mice) [11]. Integration of the L42 clone at mouse chromosome 17 was confirmed by conventional and COBRA-FISH analyses and in total 4 copies of the *Eco*RI fragment were integrated as evidenced by MLPA analysis (Figure 1B–1D). The second transgenic mouse line was generated using two overlapping PAC clones, containing the upstream *FRG1* and *FRG2* genes, an array of 12.5 D4Z4 repeat units and flanking sequences, also harboring the FSHD-permissive 4A161 haplotype and lacking exons 6 and 7 (herein referred to as D4Z4-12.5 mice) (Figure 1E) [6]. Recombination and single integration of the two PAC clones at chromosome 2 was confirmed by fiber-FISH, COBRA-FISH and MLPA analyses (Figure 1D, 1E, 1F). Thus, D4Z4-2.5 mice have D4Z4 repeat lengths that cause FSHD in humans, whereas D4Z4-12.5 mice have an array length sufficient to maintain epigenetic silencing of *DUX4* in somatic tissue in humans.

### DUX4 expression in transgenic mice

**Figure 2:**
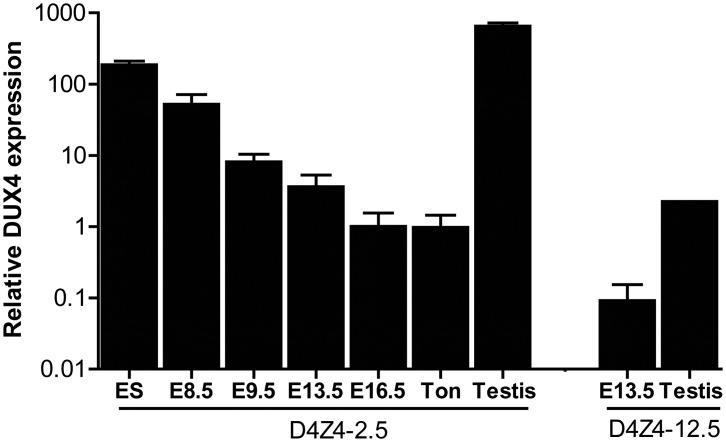
Quantative expression analysis of DUX4 transcripts from the telomeric D4Z4 unit in D4Z4-12.5 and D4Z4-2.5 mice. Quantitative RT-PCR data of DUX4 in D4Z4-2.5 embryonic stem cells (ES), in complete embryos of day E8.5, 9.5, 13,5 and 16.5, representing key myogenic developmental stages, and in adult ton = tongue, testis and in complete embryo day 13,5 and testis tissue of D4Z4-12.5 mice. Expression is normalized to the mouse reference gene Hprt and plotted in log10 scale. Error bars indicate SEM of the mean (n = 2–5).

We have previously reported that human DUX4 is expressed in stem cells of the male germline [6]. In both D4Z4-2.5 and D4Z4-12.5 mice abundant levels of DUX4 mRNA were observed in germ line tissues, most notably in testis (Figure 2). In D4Z4-2.5 mice, in situ hybridizations with specific 5′ and 3′ DUX4 probes revealed DUX4 transcripts in cells near the periphery of the seminiferous tubules (Figure S1) consistent with spermatogonia and primary spermatocytes, as has been reported for normal human testis [6]. In D4Z4-2.5 mice, abundant DUX4 mRNA levels were also detected in ES cells and early developmental stages, which were chosen based on timing of key myogenic developmental waves, showing a gradual decline during development (Figure 2) [19]–[22]. We also detected DUX4 mRNA in D4Z4-12.5 embryos, albeit at lower abundance, and in ES cells (Figure 2; Figure S3).

**Figure 3:**
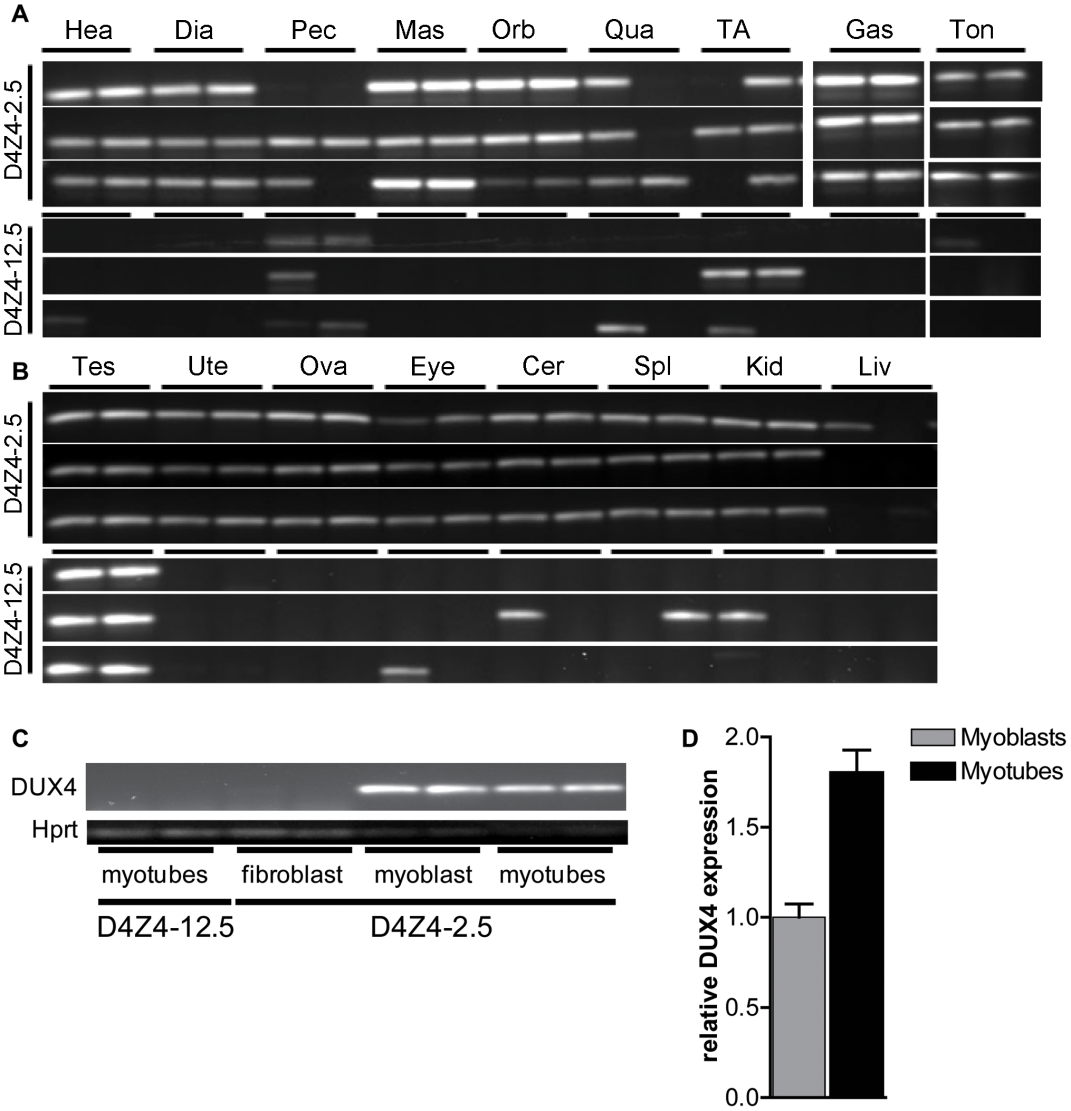
Analysis of transcriptional activity of DUX4 in a panel of tissues of D4Z4-2.5 and D4Z4-12.5 mice. DUX4 transcripts measured in 7 weeks old D4Z4-2.5 and D4Z4-12.5 mice (n = 3) in A) muscle tissue: Hea = Heart, Dia = Diaphragm, Pec = Pectoralis Mas = Masseter, Orb = Orbicularis oris, Qua = Quadriceps, TA = Tibialis anterior, Gas = Gastrocnemius, Ton = Tongue; and B) somatic non-muscle and germline tissue: Tes = Testis, Ute = Uterus, Ova = Ovarium, Eye, Cer = Cerebellum, Spl = Spleen, Kid = Kidney, Liv = Liver C) DUX4 transcripts measured in satellite-cell-derived myoblasts, myotubes and interstitial fibroblast extracted from EDL muscle of D4Z4-12.5 and D4Z4-2.5 transgenic mice. D) Quantitative RT-PCR data of DUX4 expression in D4Z4-2.5 myoblasts (n = 2) and myotubes (n = 2) 48 hours after induction of differentiation. Errors indicate SEM of the plotted mean.

We selected a panel of somatic tissues, including several skeletal muscles which are typically affected in FSHD [7], [23]. Reproducible levels of DUX4 mRNA were detected in all analyzed skeletal muscles of adult D4Z4-2.5 mice, including affected muscles of the limbs, trunk and head (Figure 3A; Figure S2). These levels were low and varied considerably between gender-matched littermates, as judged from semiquantitative analysis. Expression of DUX4 in non-muscle tissue could be expected as the candidate ortholog Dux is found to be expressed in cerebellum tissue [1]. Indeed, DUX4 transcripts were detected in non-muscle tissues, including cerebellum but with the exception of liver, where only in one mouse DUX4 could be detected once (Figure 3B; Figure S3). In D4Z4-12.5 mice, DUX4 transcripts could only be reproducibly detected in the tibialis anterior and pectoralis muscles, whereas all other somatic tissues did not show reproducible DUX4 expression (Figure 2A–2B; Figures S2 and S3). This suggests that, as seen in humans [5], [9], DUX4 is expressed variably in skeletal muscle of our transgenic mice and that decreased D4Z4 copy number contributes to inefficient DUX4 repression in somatic tissue, leading to a higher probability of expression.

**Figure 4:**
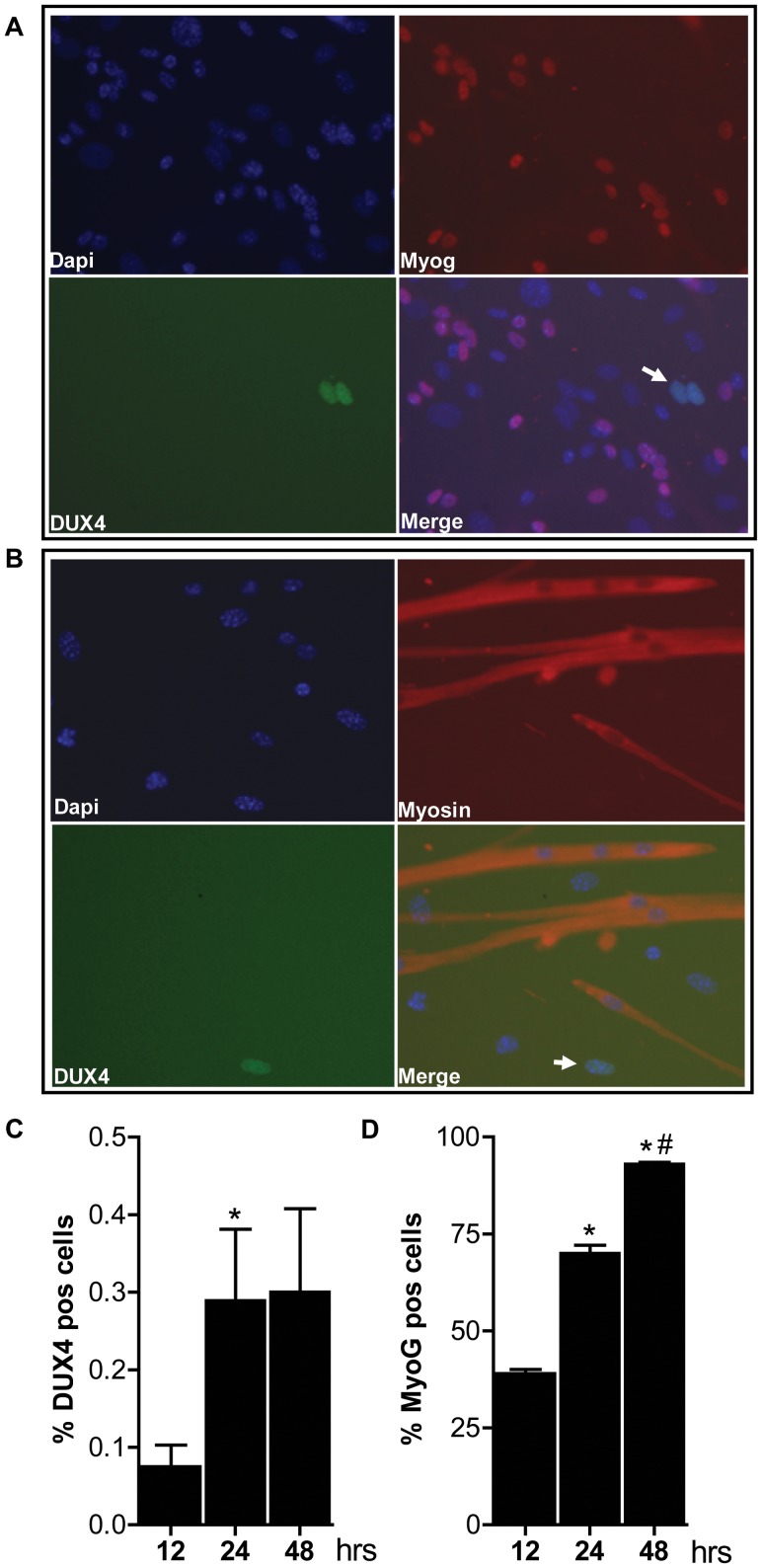
Bursts of DUX4 protein expression in differentiating D4Z4-2.5 muscle cells. Satellite-cell-derived myoblasts extracted from single EDL fibers of D4Z4-2.5 mice were differentiated for 12, 24 and 48 hrs and co-stained for DUX4 and Myog or for DUX4 and Myosin heavy chain. A) Representative DUX4 and Myog IF staining images of D4Z4-2.5 myotubes, 24 hrs after induction of differentiation, indicate absence of Myog in DUX4 expressing cells. B) Representative DUX4 and Myosin HC IF staining images of D4Z4-2.5 myotubes, 24 hrs after induction of differentiation, indicate exclusion of DUX4 positive cells from newly formed myotubes. Both DUX4 (panel C) and Myog (panel D) positive nuclei in relation to total amount of nuclei (DAPI staining) were counted during the differentiation process. C) Approximately 2∶1000 nuclei showed nuclear DUX4 staining. D) The percentage of Myog positive nuclei revealed an increase in differentiation committed cells with time. After 48 hours of differentiation almost all myoblasts are committed to differentiation. Error bars indicate stdev of the plotted mean (n = 7); *p<0,05 compared to t = 12 hrs; ^#^p<0,05 compared to t = 24 hrs.

In human FSHD muscle cell cultures, inefficient *DUX4* repression results in occasional nuclei expressing abundant amounts of DUX4. Therefore, we tested the expression of human DUX4 in D4Z4-2.5 and D4Z4-12.5 satellite-cell-derived myoblasts, both by (quantitative) RT-PCR (Figure 3C–3D) and by immunofluorescent labeling (Figure 4). In satellite-cell-derived myoblasts and differentiated myotube cultures obtained from D4Z4-12.5 mice, DUX4 transcripts and DUX4 protein could not be detected (Figure 3C and data not shown). In contrast, in satellite-cell-derived myoblasts of D4Z4-2.5 mice, DUX4 transcripts and sporadic DUX4-positive nuclei could be observed. Remarkably, all sporadic DUX4 positive nuclei were Myog negative and did not fuse into myotubes, as evidenced by co-staining with Myosin heavy chain (Figure 4A–4B). These data suggest DUX4-mediated inhibition of myoblast differentiation, as has been shown previously in zebrafish [24]. Total expression levels and frequency of DUX4-positive nuclei increased by 2–4 fold upon differentiation into myotubes (Figure 3D and Figure 4D). Satellite-cell-derived myoblasts obtained from fast-twitch (EDL) and slow-twitch (soleus) fibers from D4Z4-2.5 mice showed the same DUX4 protein expression pattern (data not shown). Of interest, interstitial fibroblasts obtained from collagenase digested D4Z4-2.5 EDL and soleus muscle did not express DUX4 transcripts (Figure 3C), indicating that the expression of DUX4 in the D4Z4-2.5 EDL and soleus is muscle cell specific.

Altogether, these data show that both the RNA and protein expression pattern of DUX4 in our D4Z4-2.5 mouse model recapitulates several features of FSHD. *DUX4* is more efficiently silenced with increasing D4Z4 copy number in our two mouse models, thereby forming a paradigm for the difference between human FSHD1 patients and healthy controls.

### Chromatin structure of D4Z4 in transgenic mice

**Figure 5:**
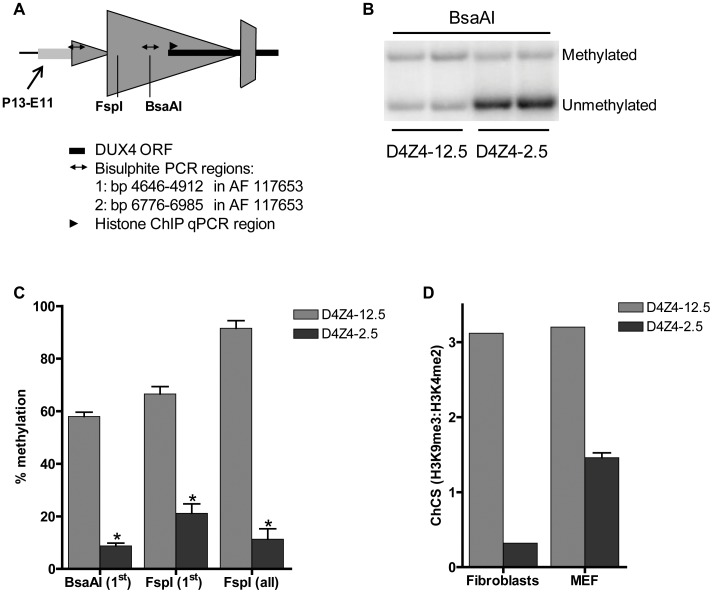
Epigenetic structure of D4Z4 in D4Z4-2.5 and D4Z4-12.5 mice. A) Schematic draw of the regions within D4Z4 where CpG and histone methylation were interrogated. B) Representative figure of a methylation sensitive Southern blot assay to quantify DNA methylation levels. Upon *Bsa*AI digestion, gel separation and blotting, two distinct bands representing the unmethylated and methylated fragment are visualized and quantified; C) Southern Blot analysis was done using two different methylation sensitive restriction enzymes, *Bsa*AI and *Fsp*I, in adult gastrocnemius muscle tissue of D4Z4-12.5 and D4Z4-2.5 mice. Both probes p13E-11 and D4Z4 were used to measure CpG methylation levels in the most proximal unit and all units, respectively. The methylation percentages of the two different CpG sites are plotted. Error bars indicate stdev of the plotted mean (n = 4 D4Z4-12.5 vs n = 5 D4Z4-2.5, *p<0.001). D) Histone methylation levels of D4Z4 in transgenic D4Z4-12.5 and D4Z4-2.5 embryonic (MEFs) and adult fibroblasts. Chromatin was precipitated with H3K4me2, H3K9me3 and control IgG antibodies. Precipitated DNA was amplified with qPCR primers amplifying the transcription start site of DUX4. Levels of H3K9me3 in relation to H3K4me2 have been plotted as the chromatin compaction score (ChCS).

Next, we studied the chromatin structure of the integrated D4Z4 repeats in order to determine whether the observed DUX4 expression patterns correlate with epigenetic differences in the FSHD and control transgenic loci. At control alleles in humans, D4Z4 is characterized by high CpG methylation levels and the co-occurrence of histone 3 lysine 9 trimethylation (H3K9me3) and histone 3 lysine 4 dimethylation (H3K4me2). FSHD alleles are epigenetically characterized by reduced D4Z4 CpG methylation and a reduced H3K9me3:H3K4me2 ratio, referred to as the chromatin compaction score (ChCS) [12], [25]–[28]. We assessed DNA methylation and the ChCS in the two mouse lines at indicated sites within the transgenic loci (Figure 5A). DNA methylation analysis using methylation-sensitive restriction enzymes followed by Southern blotting (representative blot shown in Figure 5B) showed that both the proximal and internal D4Z4 units of the array were highly methylated (60–90%) in gastrocnemius muscle of D4Z4-12.5 mice (Figure 5C), similar to unaffected individuals. In gastrocnemius muscle of D4Z4-2.5 mice, the D4Z4 units were relatively hypomethylated (10–20%; p<3.10^−5^) (Figure 5C), similar to FSHD patients and in concordance with the observed difference in DUX4 expression between the two mouse lines (Figure 3A). Similar results were obtained for quadriceps, heart, brain and liver of both transgenic lines (data not shown). Bisulphite DNA methylation analysis of different regions within D4Z4 in embryonic and adult tissues showed that D4Z4 hypomethylation in D4Z4-2.5 mice is indeed stable and uniform between tissues (Figure S4). Similar analysis in 10 month old mice showed that this epigenetic signature is stable with age (data not shown). Chromatin immunoprecipitation (ChIP) analyses in mouse fibroblasts and myoblasts showed a relative decrease in ChCS in D4Z4-2.5 compared to D4Z4-12.5 mice (Figure 5D and data not shown), similar to what was seen in patient derived human skin fibroblasts and primary myoblasts when compared to control subjects [28]. Taken together, ChIP and DNA methylation analyses indicate a relative chromatin relaxation of the D4Z4 repeats in D4Z4-2.5 mice compared to D4Z4-12.5 mice, concordant with the observed *DUX4* expression pattern, thereby accurately modeling the difference between FSHD patients and control individuals.

### Genic consequence of DUX4 expression in mouse muscle cells

In human muscle cells, DUX4 activates germline and early stem cell programs while suppressing several genes involved in the innate immune response [5]. To assess the consequence of ectopic DUX4 expression on global gene expression in mouse muscle cells, proliferating C2C12 cells were transiently transfected with a DUX4 expression vector (pCS2-DUX4) or, as a control, the empty pCS2 control vector [5]. After 24 hours, DUX4 expressing cells were enriched by FACS sorting and global gene expression changes were mapped by performing array analysis. We identified 183 significantly deregulated genes (2-fold change and FDR<0.05), of which 142 genes showed up-regulation and 41 genes showed down-regulation (Table S1). GO pathway analysis was hampered by the small number of deregulated genes, but manual inspection of the gene list revealed a considerable overlap with the deregulated genes in human myoblasts expressing *DUX4* [5]. Out of the 183 genes regulated by DUX4 in mouse C2C12 cells, 43 (23%) were previously determined to be regulated by DUX4 in human muscle cells, of which 39 changed in the same direction (Table S2).

**Figure 6:**
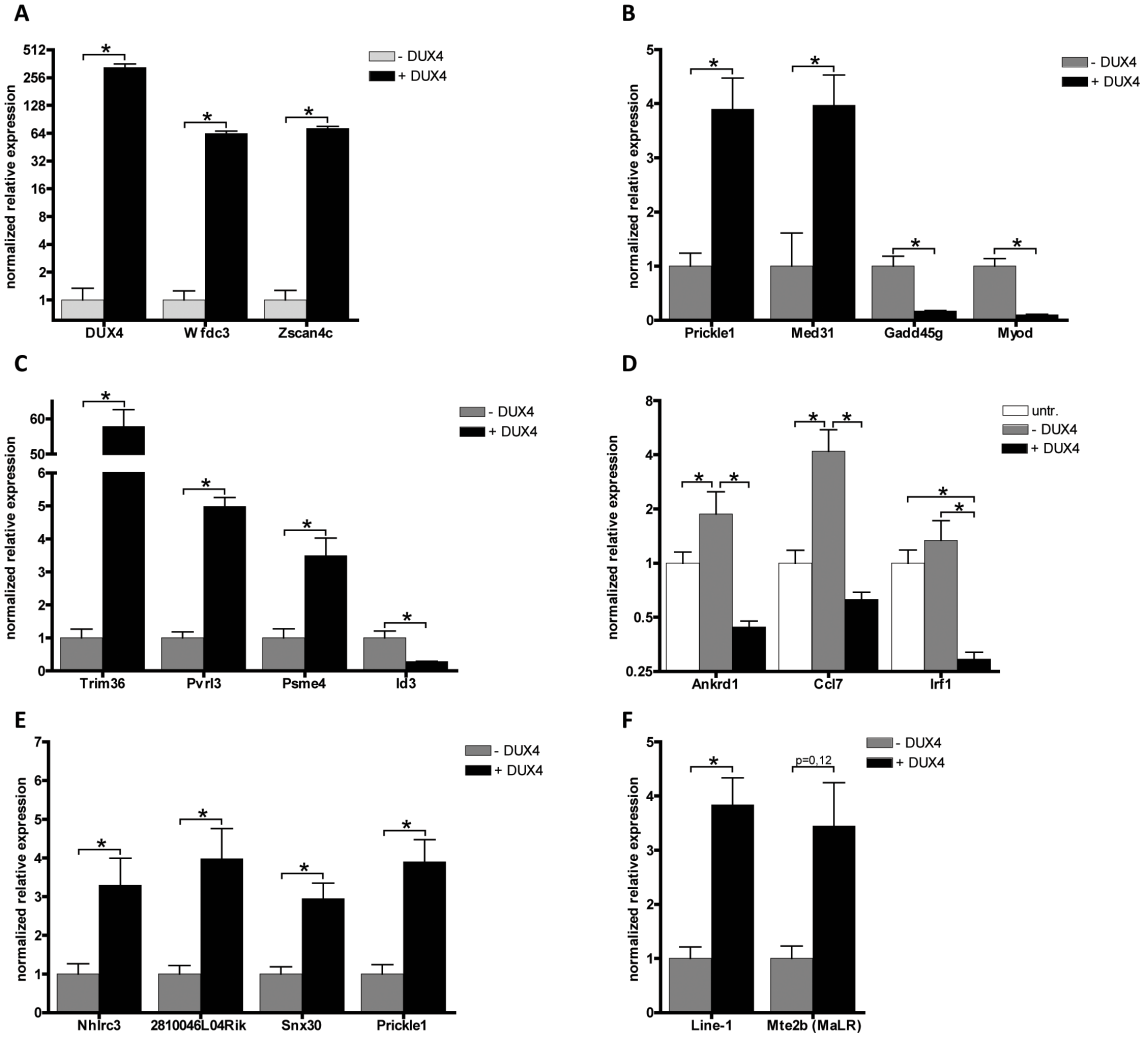
Validation of expression levels of DUX4 deregulated genes in C2C12 myoblasts. A set of deregulated genes obtained from expression array analysis was confirmed by qRT-PCR. Expression analysis of A) DUX4 and genes that are switched on by DUX4 in C2C12 cells, B) genes that respond to DUX4 in humans and mice, C) germ line and early development associated genes, D) innate immunity genes, untr = untransfected control, transfection activates innate immunity which is dampened by DUX4 expression, E) genes directly regulated by DUX4 which were identified by ChIP-seq and F) activated L1 and MaLR retrotransposons. For panel A and Mte2b in panel F, DUX4- values refer to the DUX4 depleted FACS sorted fraction, enabling proper normalization of genes switched on upon DUX4 expression. In all other panels DUX4- refers to pCS2 transfected cells. All expression levels are relative to Cyclophillin-B and normalized to DUX4- or wt conditions. Error bars indicate SEM of at least triplicate measurements, asterisks indicate p-values<0.05 based on a student's t-test (panels A, B, C, E & F) or one way ANOVA (panel D) analysis.

As in human cells, DUX4 regulated a number of germline-specific, early stem cell and innate immune response genes in the mouse C2C12 cells (Table 1). Mouse orthologs of human genes regulated by DUX4, such as *Zscan4c* and at least three orthologs of the *PRAMEF* gene family (although poorly annotated in mice) showed transcriptional activation in the presence of DUX4 (Table 1; Table S1; Figure S5). Immune modulation by DUX4 in human muscle involves a number of innate immunity related genes. Also in C2C12 cells, DUX4 regulates at least eight genes related to the innate immune response, for example *Wfdc3*, encoding a secreted peptide proposed to have antimicrobial activity [5], [29]. Using quantitative RT-PCR, we validated expression levels of *DUX4* and a selection DUX4 regulated genes in DUX4 transfected C2C12 muscle cells (Figure 6A–6D). We confirmed deregulation of genes which were switched on by DUX4 (panel A), genes which are deregulated in both human and mouse (panel B) and genes implicated in germ cell biology and early development (panel C). Deregulated genes linked to innate immunity (panel D) showed activation upon transfection, which was dampened in the presence of DUX4, as was shown in similar experiments with human myoblasts [5]. Altogether, ectopic expression of DUX4 in C2C12 cells results in deregulation of a gene set which shows overlap with DUX4 responsive genes and gene sets in human myoblasts.

**Table 1:** Deregulated genes in response to DUX4 expression in C2C12, linked to functional groups shown to be deregulated in human myoblasts upon DUX4 expression.

Germline and early development	logFc	FDR
Gm397 (Zscan4c)	1,74	1,07E-12
Trim36	1,75	1,44E-13
Pvrl3	1,15	2,01E-10
Psme4	1,08	1,65E-06
Lmo4	1,96	5,88E-12
Id1	−1,21	1,08E-09
Id3	−1,17	2,33E-09

Immune response	logFc	FDR
Wfdc3	4,44	1,00E-19
Ankrd1	−2,06	1,53E-11
Ccl7	−1,86	1,61E-11
Sema7a	−1,61	5,11E-10
Sema4b	−1,22	1,03E-08
Cxcl1	−1,56	3,55E-13
Irf1	−1,47	1,67E-10
Socs3	−1,14	3,37E-08

PRAMEF orthologues	logFc	FDR
Gm13040*	2,33	1,54E-14
Gm13043*	2,31	3,55E-13
BC080695*	2,03	5,87E-13
Gm16367	1,12	3,06E-09
Gm13119	2,21	7,84E-17

* Genes in cluster, possible duplications: >97% sequence homology.

### DUX4 can act as a transcriptional activator in C2C12

To identify genes in the mouse genome that are directly regulated by DUX4, C2C12 myoblasts were transiently transfected with pCS2-DUX4 and subjected to ChIP-seq analysis. We identified a total of 2784 peaks (P-value<10^−5^, FDR 0.02) and identified a DUX4 consensus binding sequence, highly similar to what was found in human muscle cells (Figure S5A and previously described [5]), with a strong conservation of the core TAAYYYAATCA double homeobox binding motif (Figure S6A). The genomic distribution of the identified peaks showed a slight bias for promoter sequences, as is seen for transcription factors, but not for DUX4 in human myoblasts (Figure S7) [5]. Next we identified DUX4 regulated genes (log_2_FC>[.58], FDR<0.05) that have a DUX4 binding site within a CTCF insulator domain surrounding their transcriptional start site (TSS). In this way, 91 potential direct DUX4 target genes were identified, of which 10 genes showed DUX4 enrichment within a −2 to +2 kb window from their TSS (Table S3). To validate the direct effect of DUX4, we selected 4 of these 10 direct target genes and validated their expression levels by qRT-PCR (Figure 6E). *Nhlrc3* and *2810046L04Rik* are adjacent and transcribed in opposite direction, indicating DUX4 might enhance expression from both promoters. To confirm that DUX4 indeed functions as a transcriptional activator at these sites, the DUX4 consensus binding site found at *2810046L04Rik* and *Nhlrc3* was cloned in both orientations upstream of a luciferase reporter gene and it significantly induced expression of the reporter construct when co-transfected with *DUX4* (Figure S8). Taken together, we find evidence that DUX4 can act as a transcriptional activator in mouse muscle cells as it does in human muscle cells. In addition, we identify direct DUX4 targets that might serve as suitable biomarkers in our D4Z4-2.5 mouse model of FSHD.

### Non-genic consequence of DUX4 expression in mouse muscle cells

In human cells, DUX4 has been shown to bind and activate retrotransposons, mainly of the MaLR type [5]. Our ChIP-seq analysis showed that DUX4 binds to several different types of retrotransposons also in the mouse genome. Both uniquely mappable (Table S4) and non-unique sequence reads (Table S5) show enrichment for DUX4 binding at LTR, LINE and, to a lesser extent, SINE retroelements. Quantitative RT-PCR analysis supports an upregulation of transcripts emanating from LINE-L1 and MalR (Mte2b) retrotransposons in the presence of DUX4 (Figure 6F). Like in humans, the DUX4 core TAAYYYAATCA binding motif is present in each repetitive element, although flanking nucleotides show repeat specific differences (Figure S6B–S6C) [5]. We conclude that under these experimental conditions DUX4 binds and transcriptionally activates repetitive elements in the murine genome, similar to its activity in the human genome.

### DUX4 responsive genes in D4Z4-2.5–derived muscle cell cultures, adult skeletal muscle, and embryos

**Figure 7:**
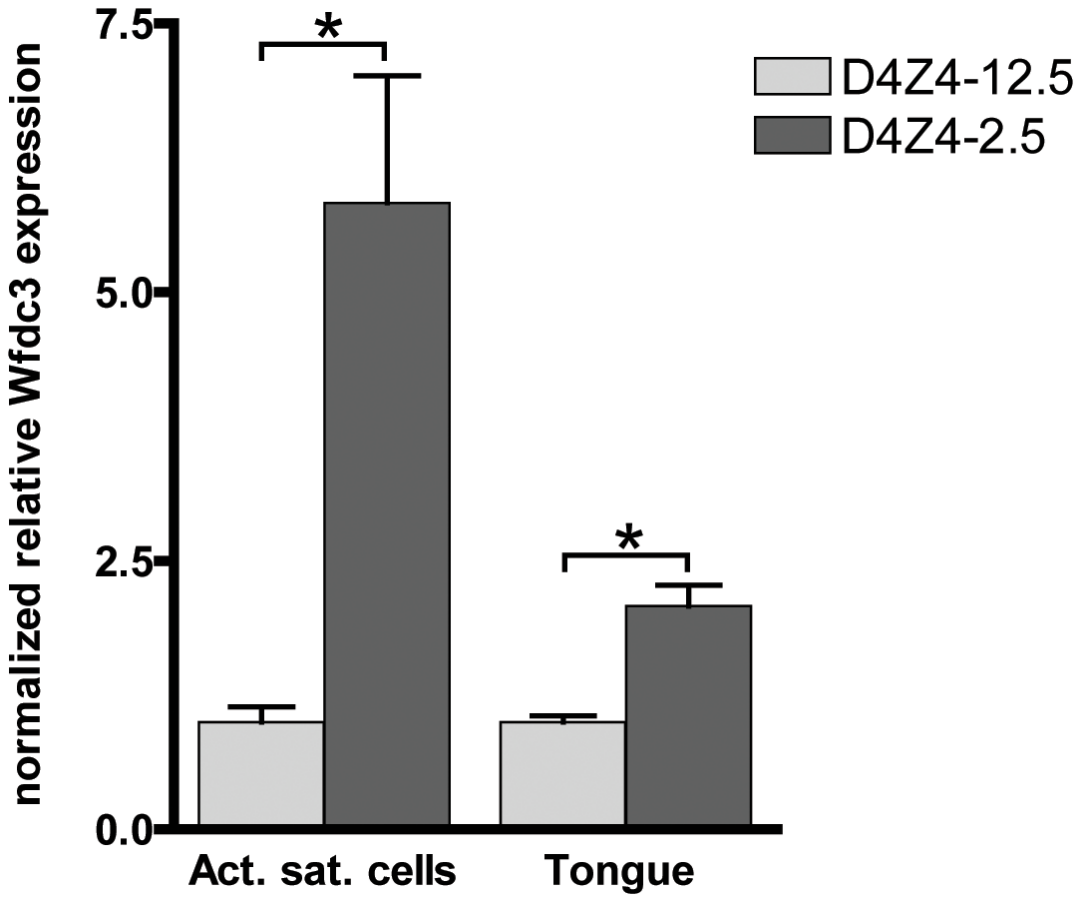
Expression of the DUX4 induced *Wfdc3* gene in myoblasts and tongue muscle of D4Z4-2.5 mice. Relative expression of Wfdc3 in satellite-cell-derived myoblast cultures from single EDL fibers (D4Z4-2.5: n = 6 and D4Z4-12.5: n = 5) and tongue tissue isolated from 7–8 weeks old mice (D4Z4-2.5: n = 6 and D4Z4-12.5: n = 6). Expression levels are relative to Cyclophilin-B and Hprt and normalized to levels in D4Z4-12.5 mice, plotted as the mean ± SEM. Asterisks indicate p<0.05 according to a independent two-tailed student's t-test.

Since DUX4 expression levels in adult D4Z4-2.5 skeletal muscle are low and only a subset of myonuclei show DUX4 protein immunoreactivity in the D4Z4-2.5 satellite-cell-derived myoblast cultures, we examined whether we could observe changes in the expression levels of DUX4 responsive genes identified by ectopic expression of DUX4 in C2C12 cells. In a set of five D4Z4-12.5 and six D4Z4-2.5 muscle cell cultures we found significant deregulation of Wfdc3 (Figure 7). In tongue muscle of D4Z4-2.5 mice, where we observed relative robust DUX4 transcript levels, again Wfdc3 levels were found to be significantly increased in D4Z4-2.5 adult mice as compared to D4Z4-12.5 adult mice (Figure 7). Since we observed relatively robust levels of DUX4 during embryogenesis, we also investigated whether we could observe the activation of Wfdc3 during this stage. In D4Z4-2.5 embryos at day E9.5 we observed robust expression of DUX4 (Figure 3) and concordantly, Wfdc3 mRNA levels increased by 2-fold compared to wildtype (WT) controls (p = 0,002) (Figure S9). Together these data showed that despite the low and variable levels of DUX4 itself, *Wfdc3* showed reproducible upregulation in D4Z4-2.5 cells and tissues, which makes it a suitable candidate for serving as a biomarker of DUX4 activity.

### Phenotype of D4Z4-2.5 mice

The phenotype of FSHD patients shows high inter- and intrafamilial variation. Next to the progressive muscle weakening, hearing loss and retinopathy are frequently observed extramuscular features [30]. The most obvious phenotype in our D4Z4-2.5 mice is the development of eye abnormalities in approximately 54% of the D4Z4-2.5 mice with an onset of around 8–12 weeks of age (Figure S10). Although variable, the mice develop a progressive keratitis of unknown etiology, possibly reflecting incomplete eyelid closure or another yet to be determined cause.

The overall morphology and histology of the limb and some head muscles appeared normal. Inducing mild muscle damage by down-hill running (eccentric activation) did not induce measurable muscle weakness and damage in D4Z4-2.5 mice. Grip strength, creatine kinase levels, Evan's blue dye uptake and proportion of central nuclei were all similar between WT and D4Z4-2.5 mice (Table S6). In addition, expression of myogenic and immunogenic markers shown to be deregulated in many mouse models for muscular dystrophies [31], were not changed in muscle of the D4Z4-2.5 mice (Table S6). Inducing muscle regeneration by cardiotoxin injection, also did not cause significant differences with respect to regeneration capacity between WT and D4Z4-2.5 mice. At 10 and 28 days after treatment, we examined the percentage of central nuclei, distribution of fiber sizes and fibrotic tissue formation. Only in the formation of fibrotic tissue, a trend towards delayed regeneration was observed as in D4Z4-2.5 a slight increase was observed at day 28 (Table S6 and data not shown).

## Discussion

*DUX4* has been implicated in FSHD pathology based on its inappropriate expression in skeletal muscle of patients with FSHD [6], [16], [17]. *DUX4* encodes a double homeobox transcription factor which activates germline, early stem cell and other programs in FSHD muscle, eventually leading to cell death [5], [32], [33]. FSHD has a complex etiology: insufficient epigenetic silencing caused by D4Z4 contraction in FSHD1 or heterozygous mutations in the chromatin modifier SMCHD1 in FSHD2 patients results in the inappropriate expression of the retrotransposed *DUX4* gene in skeletal muscle [8], [9], [14], [25], [27], [34].

In this study, we have established mouse models that recapitulate several important aspects of FSHD and control individuals with respect to the aforementioned genetic and epigenetic features of the D4Z4 macrosatellite repeat array, encoding the *DUX4* gene. Like in FSHD and control individuals, *DUX4* is expressed in the germline of D4Z4-2.5 and D4Z4-12.5 mice and, like in FSHD, D4Z4-2.5 mice show low and variable *DUX4* expression levels in somatic tissue. In FSHD patients, there is little information about somatic expression of *DUX4* in non-muscle tissue, but our studies in mice suggest that derepression of *DUX4* is not limited to skeletal muscle, consistent with the observed body-wide hypomethylation of D4Z4. In our D4Z4-12.5 mouse model we observe more efficient somatic repression of the *DUX4* locus, where *DUX4* can only be reproducibly detected in pectoralis and tibialis anterior muscle, typically affected in FSHD. Excitingly, the D4Z4-2.5 mouse model also reproduces the characteristic variegated expression pattern of DUX4 protein in FSHD muscle cell cultures: only a small sub-population of myonuclei express abundant levels of the DUX4 protein.

Thus far, several animal models over-expressing FSHD candidate genes have been produced. Some of them focused on the proximally located FSHD candidate gene *FRG1* [35]. Transgenic mice and *Xenopus laevis*, over-expressing *FRG1* either muscle-specifically or systemically, both demonstrated an abnormal musculature [36]–[38]. Nevertheless high over-expression of *FRG1* does not reflect the human FSHD expression profile and *FRG1* upregulation in FSHD muscle remains controversial [39]–[42]. *DUX4* over-expressing models have revealed the robust toxicity of DUX4 in somatic tissue, as demonstrated by massive cellular loss and abnormal development [32], [33], [37], [43]–[45]. In muscle cells, DUX4 has been shown to cause cell death and renders myoblasts hypersensitive to oxidative stress [32], [33], [43]. While these models provide insight into the potential harmful effect of DUX4 in muscle or organismal development, none of them take into account the specific endogenous expression pattern of DUX4 and its related effect.

The mouse models presented here, both carry human genomic constructs with the FSHD permissive subtelomeric region necessary for somatic DUX4 expression. Our data strongly suggests that the transcriptional profile of the *DUX4* retrogene seems to be maintained and follows the pattern observed in patients and controls. While we can find reproducible evidence of somatic derepression of *DUX4* in adult D4Z4-2.5 mice, including the variegated pattern of DUX4 positive myonuclei in cell culture, D4Z4-12.5 mice show more efficient repression of *DUX4* in their somatic tissues. This pattern of DUX4 expression suggests a locus-intrinsic property of the D4Z4 repeat array, of which the regulation is conserved between mouse and human muscle. However, we cannot rule out that some differences between the two transgenic mouse lines in the expression of *DUX4* are the consequence of different chromatin regulation at the sites of integration. We should exert some extra caution, since we only were able to generate one founder line for D4Z4-2.5 after over 450 attempts. Nonetheless, the D4Z4-2.5 mouse model is the first organismal model that can provide more insights in DUX4 regulation *in vivo* during development; e.g., why, how and when are sudden bursts of DUX4 expression in skeletal muscle regulated. This is particularly relevant since primates have lost the parental gene that retrotransposed to create the *DUX4* retrogene [18]. As it was recently demonstrated that the detrimental effects of DUX4 expression in mouse muscle can be reversed by RNA interference [46], our model may also serve well therapeutic intervention studies targeting DUX4 expression in skeletal muscle.

The FSHD specific somatic derepression of *DUX4* has been associated with changes in D4Z4 chromatin structure, characterized by decreased DNA methylation and a lower ChCS [12], [27], [28]. D4Z4-12.5 mice, containing a normal sized D4Z4 repeat array of 12.5 units, show a heterochromatic D4Z4 repeat structure comparable to human control subjects. D4Z4-2.5 mice, containing an FSHD1 sized D4Z4 array of two-and-a-half units, show a more open D4Z4 chromatin structure similar to FSHD patients. Although we cannot rule out integration-site specific effects, the CpG methylation and histone marker data of the D4Z4 arrays in both mouse lines support the model that the chromatin status of this locus is determined by a repeat length-dependent mechanism and that somatic *DUX4* repression is enforced through an evolutionary conserved mechanism of repeat-mediated silencing. The most proximal D4Z4 unit displayed lower CpG methylated than the internal units (Figure 2B and 2D–2E versus 2F–2G), a phenomenon also observed for the D4Z4 array in humans [25], [47]. In addition, we clearly demonstrated differences in DNA methylation between different CpG dinucleotides within D4Z4. Thus, even though D4Z4 is integrated at different sites within a mouse genomic background, its “human” epigenetic profile seems to be preserved. It will be of interest to study the conservation of the recently described involvement of the Polycomb/Trithorax complex in the regulation of D4Z4 and the transcripts emanating from it [48]. Our mouse model could serve as a suitable model to further study the specific epigenetic regulation as analyses are not hampered by the presence of other homologous repeat arrays or degenerate copies as is the case in human samples.

DUX4 can act as a transcription factor causing the deregulation of specific gene programs when ectopically expressed in human cells [2], [5], [14]. To better understand if the DUX4 protein can exert similar functions in the context of the mouse genome, we analyzed its effects in C2C12 cells by combining ChIP-seq with trancriptome data sets. Our ChIP-seq data showed that the core motif bound by DUX4 in human was highly conserved in the mouse, although the relative abundance of binding sites was relatively enriched for promoters in the mouse dataset [5]. In addition to regulating specific gene sets, DUX4 also binds and activates several classes of retrotransposons in the mouse genome, including Line-1 and Mte-2b, the latter one closely related to human MaLR LTRs which are specific DUX4 targets in the human genome [5]. This indicates that the primate specific retrogene *DUX4* can – at least in part – elicit a similar transcriptional response at retrotransposons in mice and raises the question whether DUX-related transcription factors in the germline are involved in retrogene biology.

Our data also showed that ectopic DUX4 expression has some similar genic consequences in mouse cells as it has in human cells. However, in contrast to humans, where DUX4 alters the expression of a large number of genes, in mice DUX4 only affects the expression levels of 183 genes, possibly reflecting the primate specificity of DUX4. This limited number of genes precluded pathway analysis, but we observed that ∼25% of genes deregulated in the mouse genome are also deregulated by DUX4 in humans, including genes involved in early development, germline biology and innate immunity. In addition, we identified a number of genes which are not shared between the mouse and human dataset, but fall in one of the aforementioned categories. Over 40% of the genes we identified to be regulated by DUX4 were also identified in the inducible DUX4 overexpression study done by Bosnakovski *et al* [32]. Combining our ChIP-seq analysis with the transcriptome analysis allowed direct DUX4 target identification and revealed that DUX4 can act as a transcriptional activator in mouse as it does in human cells.

We observed that some genes, including the early development and germline genes, become activated in mouse C2C12 muscle cells where normally these genes are not expressed. These genes may serve as good biomarkers in our mouse models for future studies tailored towards therapeutic effects of DUX4 downregulation. When studied in tissues and cell cultures isolated from our mouse models, indeed *Wfdc3* was shown to be significantly increased in D4Z4-2.5 mice compared to the D4Z4-12.5 mice. Although only a limited number of nuclei show expression of DUX4, we see robust upregulation of Wfdc3 transcription, which is also seen for the activation of target genes in human FSHD samples. The nature of this observation remains elusive, however the structure of the DUX4 transcript makes it a likely target for nonsense mediated decay, whereas its target genes are generally not.

Although we were not able to document an obvious skeletal muscle phenotype in D4Z4-2.5 mice expressing low and variable levels of *DUX4* in their muscles, we did notice a trend towards muscle weakness. The EDL muscle consists of somewhat smaller fibers and after induction of severe muscle damage by cardiotoxin injection, D4Z4-2.5 mice show a small delay in muscle regeneration. To improve therapeutic readout in our D4Z4-2.5 mouse, it will be interesting to assess DUX4 expression and muscle regeneration after multiple rounds of muscle damage, for example by crossbreeding D4Z4-2.5 with mdx mice.

Interestingly, over time more than half of the D4Z4-2.5 mice develop an abnormal eye phenotype eventually leading to blindness. Weakness of the eyelid muscles (orbicularis oculis) and thereby the difficulty in closing eyes is characteristic for FSHD. Moreover, 60% of FSHD patients also develop retinal telangiectasis, which can even lead to retinal detachment, known as Coat's syndrome [49]. Therefore it will be imperative to assess the eye pathology in our D4Z4-2.5 mouse in more detail.

In conclusion, we here report on the first transgenic mouse models which accurately model the epigenetic regulation of normal-sized and FSHD-sized D4Z4 macrosatellite repeats. While D4Z4-2.5 mice show strong overlap with the molecular phenotype of FSHD, D4Z4-12.5 mice more reflect D4Z4 regulation observed in control individuals. These mouse models will facilitate studies focusing on the *in vivo* regulation of *DUX4* and the consequences of somatic derepression of this germline transcription factor. These mouse models can also be utilized to evaluate and optimize future therapeutic strategies for FSHD.

## Materials and Methods

### Ethics statement

All animal experiments were approved by the local animal experimental committee of the Leiden University Medical Center and by the Commission Biotechnology in Animals of the Dutch Ministry of Agriculture.

### Generation and maintaining transgenic mice

D4Z4-2.5 mice were generated by microinjection of the λ42 (L42) phage into pronuclei of fertilized oocytes of B6CBAF1/J mice (Charles River Laboratories, Wilmington MA, USA). The λ42 phage contains a 13.5 kb *Eco*RI fragment encompassing the partially deleted D4Z4 locus of a patient with FSHD [50]. D4Z4-12.5 mice were generated by co-injection of the 226K22 (accession number AF146191) and 202J3 PAC clones into pronuclei of fertilized oocytes of B6CBAF1/J mice (Charles River Laboratories). PAC clone 226K22 was derived from chromosome 4A161 and isolated as described before [51]. The 202J3 PAC contained a genomic fragment extending from the *D4S2463* into the *D4Z4* repeat, including the permissive 4A161 poly-adenlyation site, but not including exon 6 and 7, and has been isolated from the RPCI-6 library (detailed on Roswell park cancer institute, Buffalo NY, USA). The transgenic mice were genotyped by PCR analysis on tail DNA. Presence of permissive haplotype containing intact polyA site distal to last D4Z4 units has been assessed by PCR followed by Sanger sequencing of the pLAM region (LGTC, Leiden, Netherlands). Primers are listed in Table S7. The transgenic mice used in these experiments were all back-crossed with C57bl6Jico mice for at least 20 generations and were bred at the animal facility of the LUMC. Mice were housed in individually ventilated cages with 12-h light–dark cycles. Standard mouse chow and water was given *ad libitum*.

### COBRA and Fiber FISH

Metaphase spread FISH analysis was done as described before [16]. COBRA-FISH was performed on D4Z4-2.5 and D4Z4-12.5 fibroblasts essentially according to the method of Szuhai et al. [52] In short, whole chromosomal painting probes (Cytocell, Adderbury, Banbury, UK) for COBRA were labeled with diethylaminocoumarin (DEAC)-, Cy3- and Cy5-ULS [reagents included in the Universal Linkage System (Kreatech Biotechnology, Amsterdam, The Netherlands), used as ratio-fluorochromes], and dGreen-ULS (used as binary fluorochrome) and combined with either biotin-16-dUTP labeled D4Z4-2.5 or 226K22 or digoxigenin-11-dUTP (Dig) labeled 202J3 (Roche Applied Science, Mannheim, Germany) by nick translation. Streptavidin-LaserPro and mouse-anti digoxigenin were applied to detect biotin and digoxigenin labeled probes, respectively. Recorded images were further processed and analyzed with an in house developed software tool (ColourProc) [52].

Fiber-FISH was done on both cultured spleen and fibroblast cells isolated from the D4Z4-2.5 and D4Z4-12.5 transgenic mice. The cells were first attached to aminosilane-coated microscope slides and then lysed to produce linear DNA fibers, which were fixed to the slide with methanol-acetic acid (3∶1). The PAC clones 226K22 and 202J3 were labeled with biotin and digoxigenin respectively. Both were hybridized simultaneously to the fiber preparations and immunocytochemically detected using Alexa Fluor 594 and fluorescein isothiocyanate (FITC), respectively. The hybridizations were analyzed using a fluorescence microscope. At least 40 fibers were assessed to obtain the order and the number of the red and green signals.

### MLPA

Probes were designed against the transgene and wild type alleles (Table S7), containing all the criteria as described in White et al [53]. Reagents for the MLPA reaction and subsequent PCR amplification were purchased from MRC-Holland (Amsterdam, The Netherlands). The MLPA reactions were performed essentially as described in Schouten et al [54]. Briefly, 200 ng of genomic DNA (concentration determined using a UV spectrophotometer) in a final volume of 5 µl was heated at 98°C for 5 minutes. After cooling to room temperature, 1.5 µl probe mix and 1.5 µl SALSA hybridization buffer was added to each sample. Next, the samples were denatured at 95°C for 1 minute and hybridized for 18 hrs at 60°C. Ligation was performed at 54°C for 15 minutes by adding 25 µl water, 3 µl buffer A, 3 µl buffer B and 1 µl ligase. The reaction was stopped by heat inactivation at 98°C for 5 minutes. PCR amplification was carried out for 30–33 cycles in a final volume of 25 µl. The MLPA primers were labeled with FAM and added with a final concentration of 200 nM. From each PCR reaction, 1,5 µl of product was mixed with 10 µl (Hi Di) formamide and 0,05 ul ROX500 size standard in a 96 well plate. Product separation was performed using capillary electrophoresis on the ABI 3700 (Applied Biosystems/Life technologies, Bleiswijk, The Netherlands). To obtain a ratio for each product, the peak height was divided by the sum of the peak heights of the wild type probes.

### Isolation of single muscle fibers and culturing of the mouse myoblasts

Mice were sacrificed by cervical dislocation and the EDL and soleus muscles were carefully dissected from tendon to tendon and digested in 0,2% collagenase (Sigma C0130)/DMEM (31966 Gibco/Life technologies) supplemented with 1% penstrep (P0781, Sigma, Zwijndrecht, The Netherlands) at 37°C for 1.45 hrs and 2 hrs respectively. The individual myofibres were dissociated by gently passing them through Pasteur pipettes with different sized apertures and then abundantly washed, as described in detail elsewhere [55]. To extract and expand the satellite cell pool, muscle fibers were cultured on matrigel (354230, BD biosciences, Breda, The Netherlands) coated 6-wells plate in DMEM 31966 supplemented with 30%FBS, 10%HS, 1%CEE and 2.5 ng/ml FGF, 1% pen-strep (all Gibco/Life technologies), 150 fibers per well. After 3 days, the fibers were detached and removed. The attached myoblasts were trypsinized, counted and plated for further analysis. To induce differentiation into myotubes, serum-rich medium was replaced with serum-poor medium (DMEM 31966), supplemented with 2% HS and 1% pen-strep, 48 hrs after plating. After 48 hours of differentiation cells were either fixed for immunofluorescence or lysed to isolate RNA.

### Generation MEFs and adult skin fibroblasts

Mouse embryonic fibroblasts were generated from at embryonic stage E13.5. First, embryos were dissected out of the uterine horns, rinsed with 70% EtOH and washed in PBS (14190-169 Gibco/Life technologies). Next, the embryos were separated from the placenta and surrounding membranes. Tails of embryos were cut and used for genotyping. Next, the dark red organs were removed and embryos were finely minced and suspended in 1,5 ml of trypsin-EDTA (25300-096, Gibco/Life technologies) for 15 minutes at 37°C with gentle shaking. Trypsin was inactivated by the addition of 2 ml MEF medium: DMEM high glucose (41966-052, Gibco/Life technologies) supplemented with 10% FCS, 1% L-glutamine (25030-024, Gibco/Life technologies) and 1% pen/strep. Upon centrifugation (5 minutes, 1200 rpm), the minced and trypsinized cell pellet was suspended in MEF medium and plated on gelatin coated culture dishes.

Adult skin fibroblasts were generated by dissecting skin tissue from the belly of 5 months old mice. The skin of each mice was dissociated overnight at RT in 2 ml dispase/collagenase mix, containing 2 mg dispase (17105-041, Invitrogen/Life Technologies), 2 mg collagenase (C-9891, sigma), 0,04 ml pen/strep, 0,04 ml glutamine, 0,25 mg fungizone and 0,2 mg gentamicine (all Gibco/Life technologies). Next day, cells were centrifuged (5 minutes, 1200 rpm) and resuspended in 6 ml MEF medium and plated in T25 culture flasks.

### Immunofluorescence

For co-IF staining of DUX4, myogenin (Myog) and myosin heavy chain, cells were fixed in 2% paraformaldehyde for 7 minutes at room temperature and then washed twice with PBS. Cells were permeabilized with 1% Triton X-100 (Sigma) in PBS for 10 minutes at room temperature with gentle rocking. Primary rabbit-DUX4 antibody directed against the C-terminal region of DUX4 (E5-5; 1∶100) [56], Myog (1∶100, Dako North America, Carpinteria CA, USA) and MF-20 anti myosin heavy chain (1∶100, Developmental studies hybridoma bank, Univ. of Iowa, Iowa city, IA USA) were diluted in PBS and cells were incubated overnight at 4°C with the first antibody. After washing three times in PBS, followed an incubation with diluted Alexa 488 conjugated donkey anti-rabbit and Alexa 594 conjugated donkey anti-mouse (A21206, A21206, 1∶500, Invitrogen/Life technologies) for one hour, gently rocking in the dark. Next, cells were washed three times with PBS-0,025% TWEEN before they were mounted on microscope slides using Aqua Poly/Mount (PolySciences, Warrington PA, U.S.A.) containing 500 ng/ml DAPI. Stained cells were analyzed on a Leica DMRA2 microscope (Leica microsystems, Wetzlar, Germany).

### gDNA, RNA isolation, and cDNA synthesis

gDNA isolation from different tissues of both D4Z4-2.5 and D4Z4-12.5 mice was carried out using the Genomic DNA from tissue kit (740952, Machery-Nagel, Düren, Germany) following manufacturers instruction. Total RNA was isolated from tissue using miRNeasy kit (217004, Qiagen, Venlo, The Netherlands), including a DNase treatment, according to the instructions of the manufacturer. For C2C12 expression analysis, RNA was isolated using the RNeasy microkit (74004, Qiagen). Both DNA and RNA concentrations were determined using a Nanodrop ND-1000 spectrophotometer (Thermo Scientific, Wilmington DE, USA). The quality of the RNA was assessed by using a RNA 6000 nanochip on an Agilent 2100 BioAnalyzer (Agilent Technologies Netherlands BV, Amstelveen, The Netherlands) and RIN scores>9 were obtained. cDNA was synthesized using 1–3 µg total RNA using the Revert Aid H Minus first strand cDNA synthesis kit using oligo dT primed primers (Fermentas/Thermo scientific, St. Leon-Rot, Germany) according to the manufacturer's instructions. The cDNA was subsequently treated with 0.5 U RNaseH for 20 min at 37°C and total cDNA was diluted in 50 ul water. For all C2C12 expression studies, polydT primed cDNA was synthesized using 300 ng of RNA using the Omniscript RT kit (205111, Qiagen) according to manufacturer's instructions. RT was done at 50°C and cDNA was diluted to a total volume of 100 µl.

### DNA methylation analysis by Southern blotting

Methylation levels of individual CpGs was determined by Southern blot analysis using the methylation-sensitive restriction enzymes *Bsa*AI and *Fsp*I as described before [12]. In short, 5 µg of genomic DNA was digested with the restriction enzymes *Eco*RI and *Bgl*II and either *Bsa*AI (NEB, Ipswich MA, USA) or *Fsp*I (NEB). All digestions were performed according to the manufacturer's instructions. After digestion, DNA was separated by standard linear gel electrophoresis (0.8%) followed by Southern blotting of the DNA on a hybond-XL membrane (GE Healthcare) and hybridization with the radioactive labeled probe p13E-11 (D4F104S1) or D4Z4 to determine the methylation in the proximal D4Z4 repeat unit and all internal units respectively. Hybridizations were performed for a minimum of 16 hours at 65°C in 0.125 M Na_2_HPO_4_ (pH 7.2), 0.25 M NaCl, 1 mM EDTA, 7% SDS and 100 µg/ml denatured fish sperm DNA (Roche). After hybridization, membranes were consecutively washed with 2xSSC/0.1%SDS, 1xSSC/0.1%SDS and 0.3xSSC/0.1%SDS. Finally, the membranes were exposed to a phospho-imager screen and signal intensities were quantified with ImageQuant software (Amersham/GE healthcare).

### Quantitative methylation analysis by bisulphate converted DNA

Bisulphite treatment was performed with the EZ DNA Methylation kit (Zymo Research, Irvine CA, USA) and bisulphite primers were designed with MethPrimer software [57]. Primer sequences are listed in Table S7. PCR was performed in a final volume of 25 µl containing 250 µM dNTPs, 1X Supertaq PCR Buffer (HT Biotechnology Ltd, Cambridge UK), 10 pM of each primer and 1 U Silverstar DNA polymerase (Eurogentec, Maastricht, The Netherlands). Cycling conditions: 94°C for 15 min followed by 40 cycles of 94°C for 40 s, 58°C for 40 s, and 72°C for 40 s, and a final extension step of 15 min at 72°C. PCR products were purified directly or by gel extraction using the NucleoSpin Gel and PCR Clean-up kit (Machery-Nagel) and subjected to Sanger sequencing (LGTC). Quantitative methylation ratios were calculated from sequence traces using the ESME software, including appropriate sequence quality control, normalization of signals, correction for incomplete bisulphite conversion, and mapping of positions in the trace file to CpGs in reference sequences [58].

### Histone chromatin immunoprecipitation

ChIP experiments were based on the protocol described by Nelson *et al.* with some modifications [59]. In brief, subconfluent cell cultures were crosslinked in 1% formaldehyde for 10 minutes and the reaction was quenched for 5 minutes with glycine at a final concentration of 125 mM. Crosslinked cells were lysed and chromatin was sheared in a sonicator bath (Bioruptor UCD-20, Diagenode, Liège, Belgium) for 4–6 consecutive rounds of 10 minutes at maximum output and 15 seconds on/off cycles. Shearing was analyzed by phenol-chloroform extraction of DNA and agarose gel electrophoresis. All chromatin samples had a DNA size range between 200–2000 bp with a peak around 200 bp. Per reaction 3 µg (DNA content) of chromatin was precleared with blocked sepharose A beads (GE healthcare, Diegem, Belgium) and incubated overnight at 4° with antibodies: rαH3K9me3 (17–625, Millipore, Billerica MA, USA; 5 µl/rxn) rαH3K4me2 (07–030, Millipore; 5 ul/reaction) and total IgG (Millipore; 5 µl/rxn). IP was done with 20 µl sepharose A beads/reaction and washing was according to the online available Millipore ChIP protocol. DNA was isolated using Chelex resin and diluted 1∶1 for qPCR analysis [59]. Relative abundance, IgG corrected and input normalized, at D4Z4 was determined with previously published primers [27].

### Quantitative PCR

All quantative RT-PCR analysis were performed in duplicate using SYBR green mastermix on the MyIQ or CFX96 system (Bio-Rad, Veenendaal, The Netherlands) using 0.5–0.75 pM of each primer in a final volume of 10–15 µl per reaction. For gene expression analysis 2–5 µl of diluted cDNA and for ChIP analysis 5 µl of 1∶1 diluted ChIP DNA was used per reaction. Cycling conditions: initial denaturation step at 95°C for 3 min, followed by 35–40 cycles of 10–15 s at 95°C and 45 s at primer Tm (Table S7). Specificity of all reactions was monitored by standard gel electrophoresis and/or melting curve analysis: initial denaturation step at 95°C, followed by 1 min incubation at 65°C and sequential temperature increments of 0.5°C every 10 s up to 95°C. All primer sets were designed using Primer3 software and, for cDNA analysis, spanned at least one intron. Results were analyzed using iQ5/Bio-Rad CFX manager version 2.0 (Bio-Rad). For cDNA, relative expression was calculated, using Cyclophilin, Hprt or Gapdh as a reference gene (indicated) for cDNA input, using the CFX manager software. For ChIP, relative quantification was done by background subtraction based on the signal in the normal IgG ChIP. Normalization was done using the relative abundance of the product in DNA isolated from ChIP input samples.

### C2C12 culture, transfection, and cell sorting

For C2C12 cultures, plates and dishes were coated with collagen (Purecol, Advanced Biomatrix, San Diego CA, USA) 1∶30 diluted in MiliQ, for 1 hr at 37°C and then dried for at least 30 minutes. Before plating the cells, plates and dishes were washed with 1xPBS. C2C12 cells were maintained subconfluent in DMEM (11880, Gibco/Life technologies) supplemented with 20% FCS, 1% pen/strep, 1% glucose and 1% glutamax (all Gibco/Life technologies). Transfections were performed using lipofectamine reagent, combined with plus reagent (both Invitrogen/Life Technologies) according to manufacturer's instructions in 12/6 well plates, 9 or 20 cm dishes with 0.6/1.5, 5 or 12 µg total plasmid DNA, respectively. To enrich for DUX4 expressing cells used for gene expression analysis, pCS2-DUX4 [5] or the empty pCS2 backbone were equimolarly co-transfected with pEGFP-C1 (Life technologies) and then sorted on a FACS aria cell sorter II (BD biosciences). In short, the living single cells were first gated based on forward and side scatter and then sorted by gating for GFP. Both the GFP enriched and depleted fractions were collected in PBS supplemented with 1% FCS, spun down and stored at −80°C.

### Expression array analysis

Global gene expression changes upon DUX4 expression were obtained with illumina MouseWG-6 v2.0 expression arrays (Illumina, San Diego CA, USA). RNA from FACS sorted C2C12 transfected with pCS2-DUX4 were compared with pCS2 backbone transfected cells in triplicate. Labeling of RNA, hybridization of the arrays and primary data analysis were carried out by ServiceXS (Leiden, The Netherlands) according to manufacturer's instructions. Probe intensities were corrected and normalized using the lumiExpresso function from the lumi Bioconductor package [60] with default options. If a probe was not present in any of the 6 arrays according to lumi's “detectionCall” function, we removed it from further consideration. Differentially expressed probes were then identified using the limma Bioconductor package [61] and p-values were adjusted to account for multiple testing using Benjamini and Hochberg's method [62].

### DUX4 ChIP–seq analysis

Crosslinking, generation of chromatin and ChIP were performed as indicated above. In brief, chromatin of wt and DUX4 transfected C2C12s containing 12 µg DNA was precleared with IP beads and incubated o/n at 4°C with the rabbit polyclonal MO489 antibody directed against the C-terminus of DUX4 [5]. After washing 6 times in ChIP buffer, immunoprecipitated ChIP DNA was isolated and purified with a standard phenol extraction. Sequencing and sample preparation were done with the Illumina genome analyzer following manufacturer's instructions. Data analysis was performed as previously described [5]. Briefly, sequences were extracted by GApipeline-0.3.0. Reads mapping to the X and Y-chromosomes were excluded from our analysis. Duplicate sequences were discarded to minimize effects of PCR amplification and each read was extended in the sequencing orientation to a total of 200 bases to infer the coverage at each genomic position. Peak calling was performed by an in-house developed R package, which models background reads by a negative binomial distribution as previously described [63]. To identify the DUX4 consensus binding sequence, we applied an in-house developed Bioconductor package motifRG for discriminative de novo motif discovery as previously described [64], [65].

For supporting materials and methods concerning supplemental data, please refer to Text S1.

## Supporting Information

Figure S1 In situ hybridization to detect DUX4 mRNA in D4Z4-2.5 mouse testis. Frozen sections of control mouse testes (A,D) and D4Z4-2.5 mouse testes (B–C, E–F) hybridized with antisense RNA probes for the 5′ (A–C) or 3′ (D–F) regions of human *DUX4*. C and F show magnifications of indicated regions in B and E respectively. Control testes show no staining, whereas D4Z4-2.5 testes show staining for *DUX4* in large round cells near the periphery of the tubules (arrows), likely in spermatogonia and spermatocytes.
(TIF)

Figure S2 DUX4 Expression analysis in muscles of D4Z4-2.5 and D4Z4-12.5 mice. DUX4 RT-PCR analysis in duplicate of muscle tissues of adult A) D4Z4-2.5 (n = 3) and B) D4Z4-12.5 (n = 3) mice in Hea = Heart, Dia = Diaphragm, Pec = Pectoralis Mas = Masseter, Orb = Orbicularis oris, Qua = Quadriceps, TA = Tibialis anterior, Gas = Gastrocnemius, Ton = Tongue. Hprt was used as control for RNA integrity.
(TIF)

Figure S3 DUX4 Expression analysis in non-muscle tissue of D4Z4-2.5 and D4Z4-12.5 mice. DUX4 RT-PCR analysis in duplicate of non-muscle tissues of adult A) D4Z4-2.5 (n = 3) and B) D4Z4-12.5 (n = 3) mice in Tes = Testis, Ute = Uterus, Ova = Ovarium, Eye, Cer = Cerebellum, Spl = Spleen, Kid = Kidney, Liv = Liver. Hprt was used as control for RNA integrity. C) analysis of D4Z4-2.5 testis cDNA generated without reverse transcriptase. D) Expression of DUX4 in D4Z4-12.5 derived ES cell clones. Gapdh was used as a control for RNA integrity.
(TIF)

Figure S4 CpG methylation analysis of D4Z4 in D4Z4-2.5 and D4Z4-12.5 mice. Upper panel: Schematic draw of the regions within D4Z4 where CpG and histone methylation were interrogated. A–D) Methylation levels of individual CpGs in the first partial D4Z4 unit (A,B) or upstream of the *DUX4* ORF (C,D) were analyzed in whole D4Z4-12.5 and D4Z4-2.5 embryos (E13,5) (A,C) and gastrocnemius muscle tissue of 2 month old D4Z4-12.5 and D4Z4-2.5 mice (B,D). Plotted as mean ±stdev. of n = 8 D4Z4-12 vs n = 5 D4Z4-2.5 mice, *p<5.10^−10^.
(TIF)

Figure S5 Intensity plot of array probes of C2C12+pCS2-DUX4 versus C2C12+pCS2. Normalized, log transformed mean intensities (triplicates) of C2C12+pCS2 are plotted against C2C12+pCS2-DUX4 per probe. Black dots indicate significantly deregulated probes. Circled probes indicate DUX4 activated genes, with very low intensities (≤7.5) in pCS2 transfected cells.
(TIF)

Figure S6 Consensus binding site sequence sequences of DUX4 at different genetic contexts. Position weight matrices of the DUX4 consensus binding sequence at A) unique binding sites, B) MaLR retrotransposons and C) L1 retrotransposons. The core double homeobox is conserved at all regions, flanking nucleotides show context specific variation.
(TIF)

Figure S7 Distribution of DUX4 binding sites in the mouse genome. Relative distribution of ChIP peaks is displayed as the number of peaks per kb of total genomic sequence for each context. DUX4 shows a slight promoter bias as seen for transcription factors, but not for DUX4 in human myoblasts.
(TIF)

Figure S8 Luciferase reporter assays using a direct DUX4 target site. Normalized relative luciferase activity of the *Nhlcr3* and *2810046L04Rik* bidirectional DUX4 binding site in forward (Fw) and reverse (Rev) orientation in the absence (pCS2) or presence (pCS2-DUX4) of DUX4. Below a schematic overview of the DUX4 binding site is shown. All values are first normalized to pGL3 basic expression levels with or without DUX4. Next, the levels in DUX4 negative cells were set to 1. Error bars indicate SEM of quadruple measurements. Asterisks indicate p<0.05 according to a student's t-test.
(TIF)

Figure S9 Expression analysis of the DUX4 target *Wfdc3* in E9,5 embryos. Wfdc3 shows significant upregulation in D4Z4-2.5 embryos compared to wt controls (n = 6). Expression levels are normalized to the mouse reference gene *Hprt* and are plotted as the mean ± SEM. Asterisks indicate p<0.05 according to a student's t-test.
(TIF)

Figure S10 Keratitis in D4Z4-2.5 mice at different ages. Representative pictures of eyes of WT (A,C,E,G) and D4Z4-2.5 (B,D,F,H) mice at 11 weeks (A&B), 17 weeks (C&D), 27 weeks (E&F) and 1,5 years (G&H) of age.
(TIF)

Table S1 Deregulated genes in response to ectopic DUX4 expression in C2C12 myoblasts.
(XLS)

Table S2 DUX4 induced deregulated genes overlapping between C2C12 and human myoblasts.
(XLS)

Table S3 Identified direct targets of DUX4 in C2C12 myoblasts.
(PDF)

Table S4 Unique DUX4 binding sites in transposable elements in C2C12 myoblasts.
(XLS)

Table S5 DUX4 binding sites in transposable elements in C2C12 myoblasts.
(XLS)

Table S6 Overview of the histological and functional tests performed to investigate muscle integrity and performance in D4Z4-2.5 mice.
(XLS)

Table S7 List of primers and corresponding sequences.
(PDF)

Text S1 Supporting Materials and Methods and References.
(PDF)
