# Supplementary figures and images for: Intrinsic Epigenetic Regulation of the D4Z4 Macrosatellite Repeat in a Transgenic Mouse Model for FSHD
Source: PLoS Genet. 2013 Apr 4;9(4):e1003415. doi: 10.1371/journal.pgen.1003415 (PMC3616921; doi:10.1371/journal.pgen.1003415)

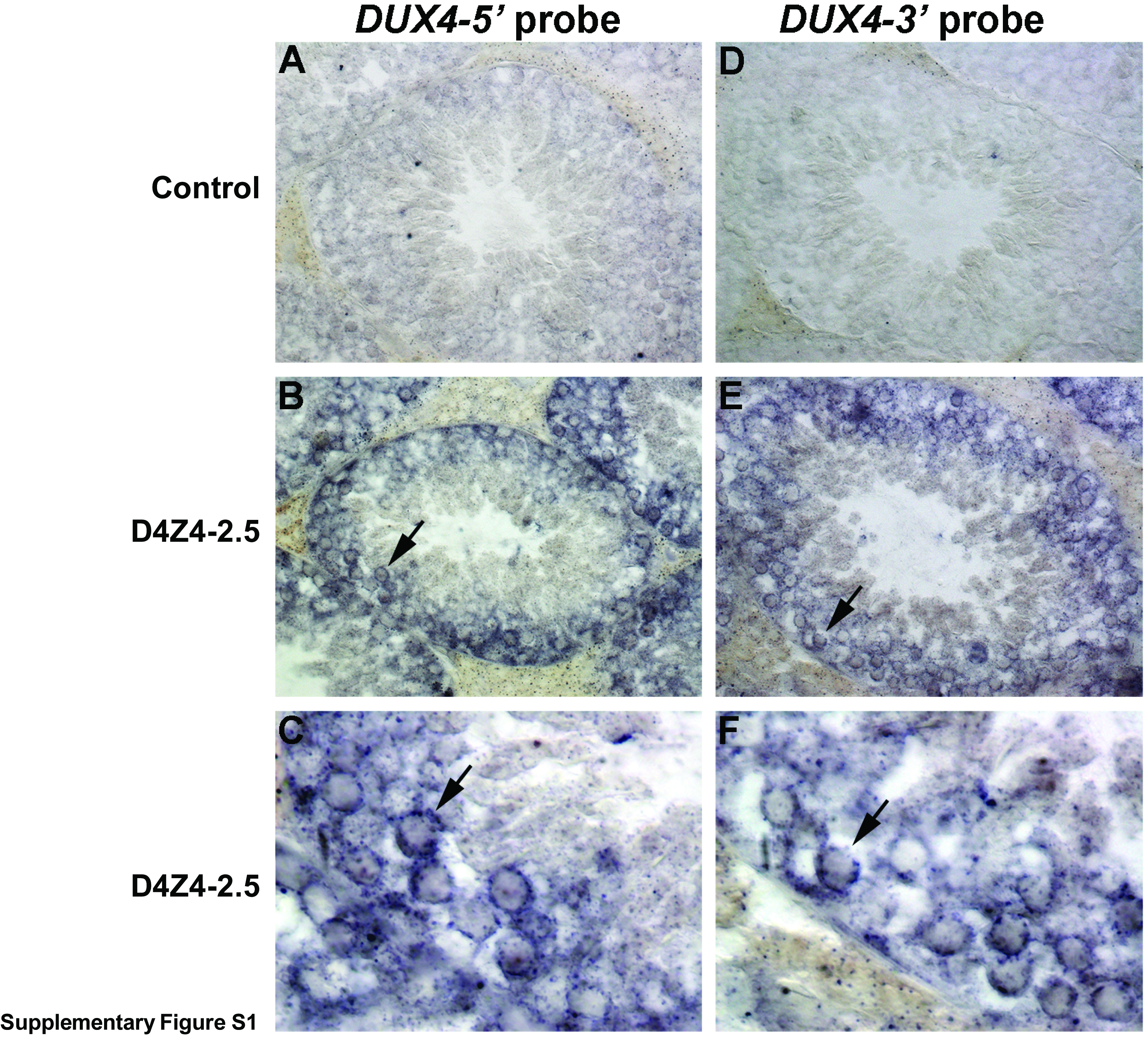

Supplement: Figure S1 — In situ hybridization to detect DUX4 mRNA in D4Z4-2.5 mouse testis. Frozen sections of control mouse testes (A,D) and D4Z4-2.5 mouse testes (B–C, E–F) hybridized with antisense RNA probes for the 5′ (A–C) or 3′ (D–F) regions of human DUX4. C and F show magnifications of indicated regions in B and E respectively. Control testes show no staining, whereas D4Z4-2.5 testes show staining for DUX4 in large round cells near the periphery of the tubules (arrows), likely in spermatogonia and spermatocytes. (TIF) [file pgen.1003415.s001.tif]

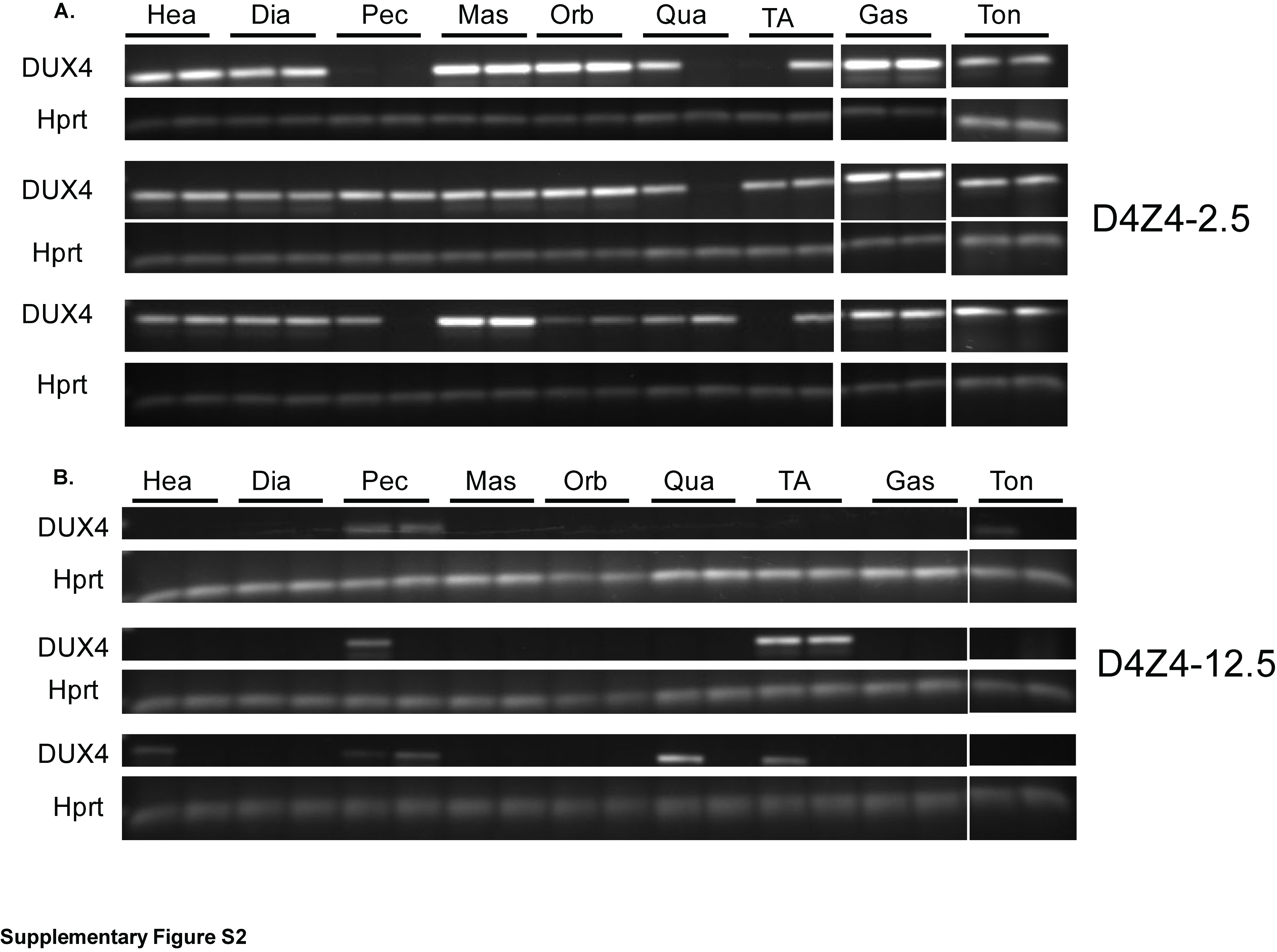

Supplement: Figure S2 — DUX4 Expression analysis in muscles of D4Z4-2.5 and D4Z4-12.5 mice. DUX4 RT-PCR analysis in duplicate of muscle tissues of adult A) D4Z4-2.5 (n = 3) and B) D4Z4-12.5 (n = 3) mice in Hea = Heart, Dia = Diaphragm, Pec = Pectoralis Mas = Masseter, Orb = Orbicularis oris, Qua = Quadriceps, TA = Tibialis anterior, Gas = Gastrocnemius, Ton = Tongue. Hprt was used as control for RNA integrity. (TIF) [file pgen.1003415.s002.tif]

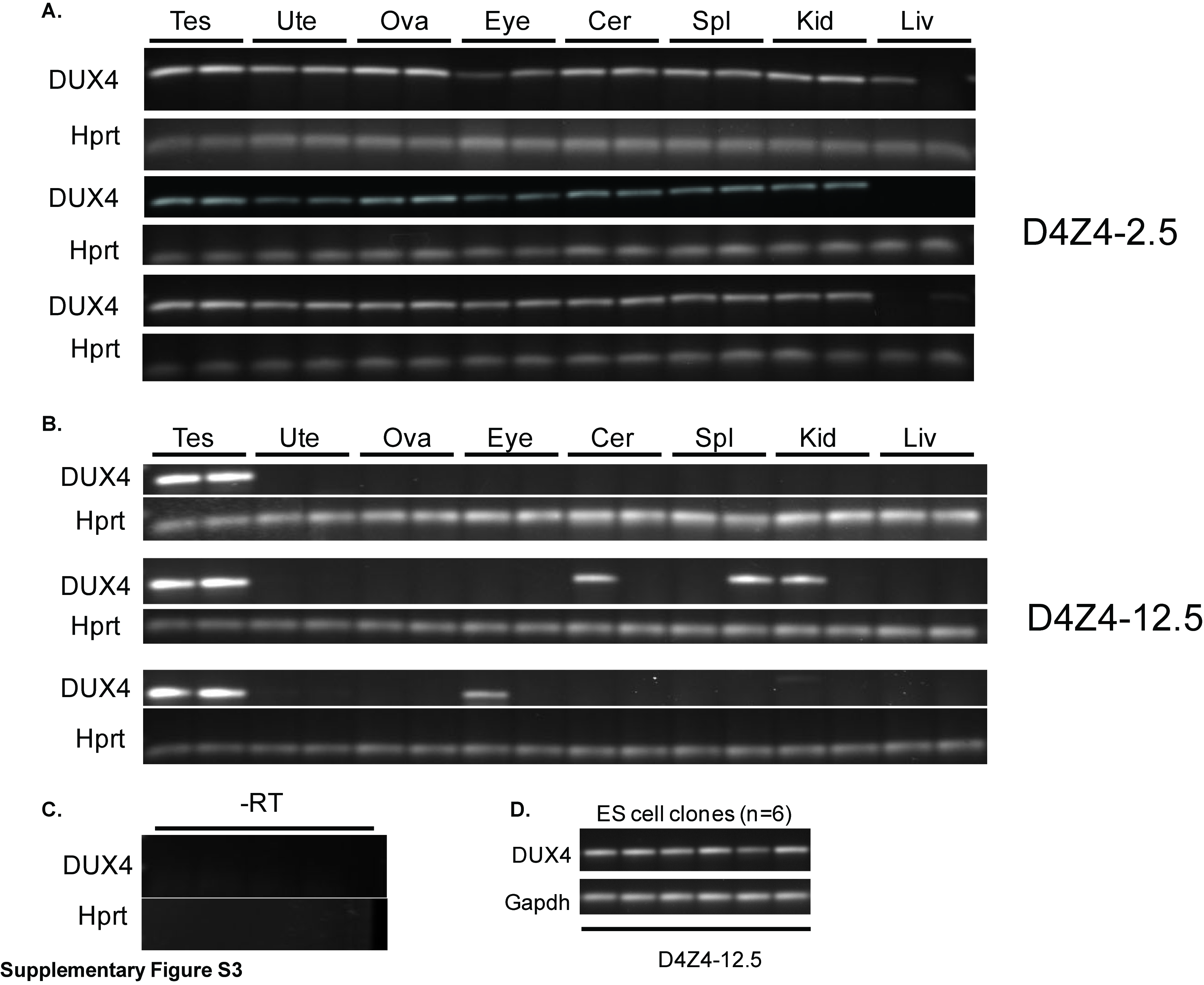

Supplement: Figure S3 — DUX4 Expression analysis in non-muscle tissue of D4Z4-2.5 and D4Z4-12.5 mice. DUX4 RT-PCR analysis in duplicate of non-muscle tissues of adult A) D4Z4-2.5 (n = 3) and B) D4Z4-12.5 (n = 3) mice in Tes = Testis, Ute = Uterus, Ova = Ovarium, Eye, Cer = Cerebellum, Spl = Spleen, Kid = Kidney, Liv = Liver. Hprt was used as control for RNA integrity. C) analysis of D4Z4-2.5 testis cDNA generated without reverse transcriptase. D) Expression of DUX4 in D4Z4-12.5 derived ES cell clones. Gapdh was used as a control for RNA integrity. (TIF) [file pgen.1003415.s003.tif]

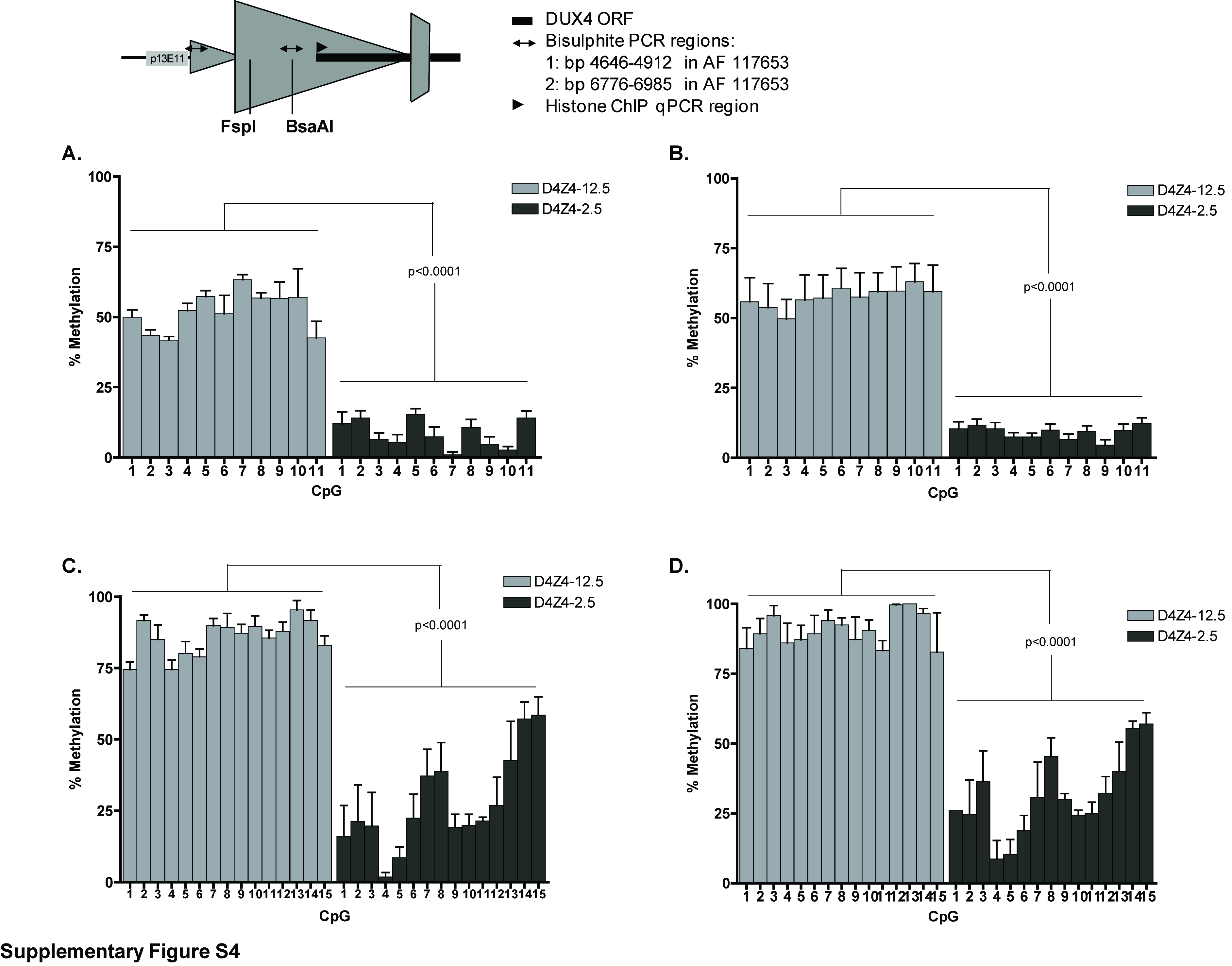

Supplement: Figure S4 — CpG methylation analysis of D4Z4 in D4Z4-2.5 and D4Z4-12.5 mice. Upper panel: Schematic draw of the regions within D4Z4 where CpG and histone methylation were interrogated. A–D) Methylation levels of individual CpGs in the first partial D4Z4 unit (A,B) or upstream of the DUX4 ORF (C,D) were analyzed in whole D4Z4-12.5 and D4Z4-2.5 embryos (E13,5) (A,C) and gastrocnemius muscle tissue of 2 month old D4Z4-12.5 and D4Z4-2.5 mice (B,D). Plotted as mean ±stdev. of n = 8 D4Z4-12 vs n = 5 D4Z4-2.5 mice, *p<5.10−10. (TIF) [file pgen.1003415.s004.tif]

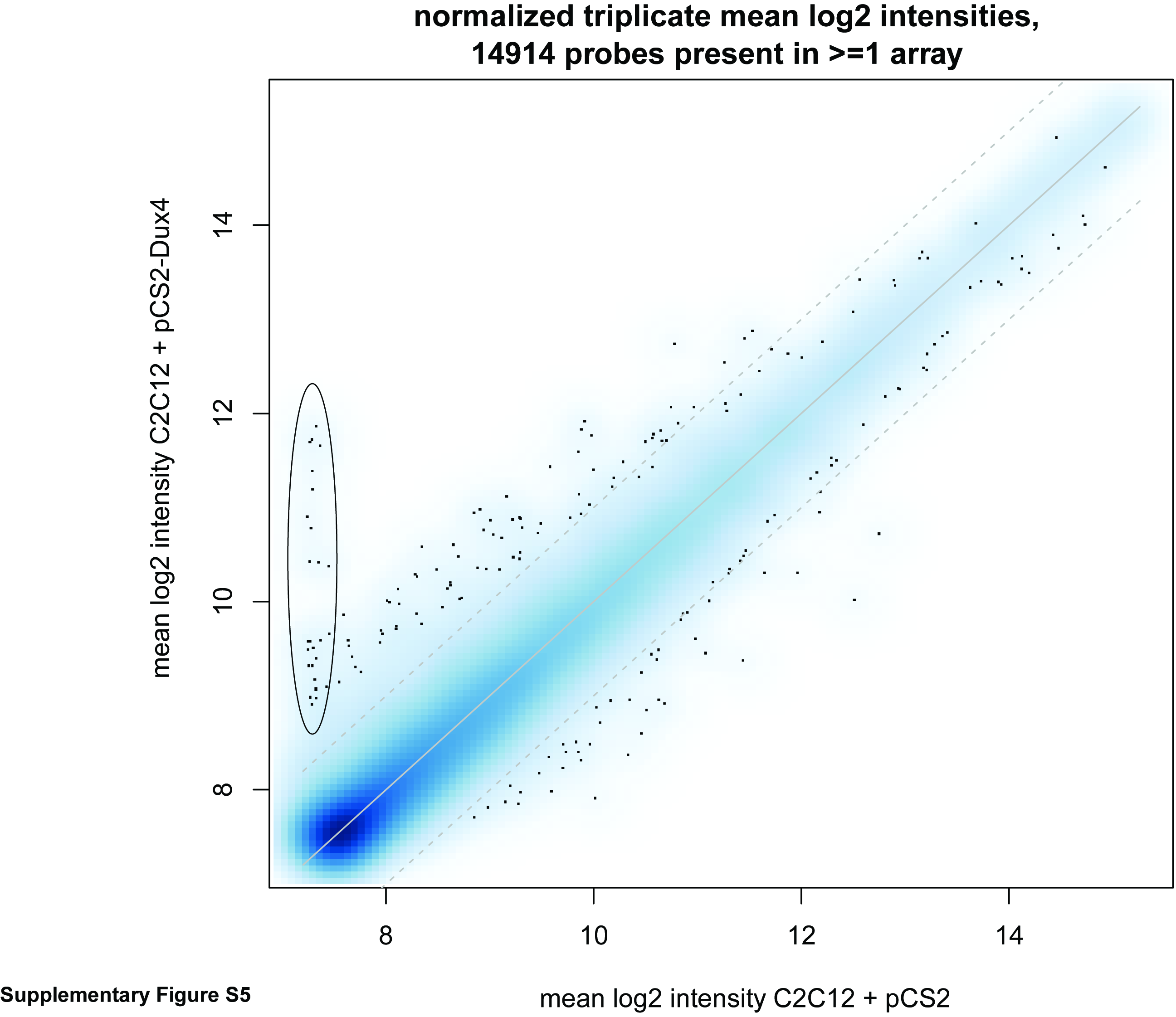

Supplement: Figure S5 — Intensity plot of array probes of C2C12+pCS2-DUX4 versus C2C12+pCS2. Normalized, log transformed mean intensities (triplicates) of C2C12+pCS2 are plotted against C2C12+pCS2-DUX4 per probe. Black dots indicate significantly deregulated probes. Circled probes indicate DUX4 activated genes, with very low intensities (≤7.5) in pCS2 transfected cells. (TIF) [file pgen.1003415.s005.tif]

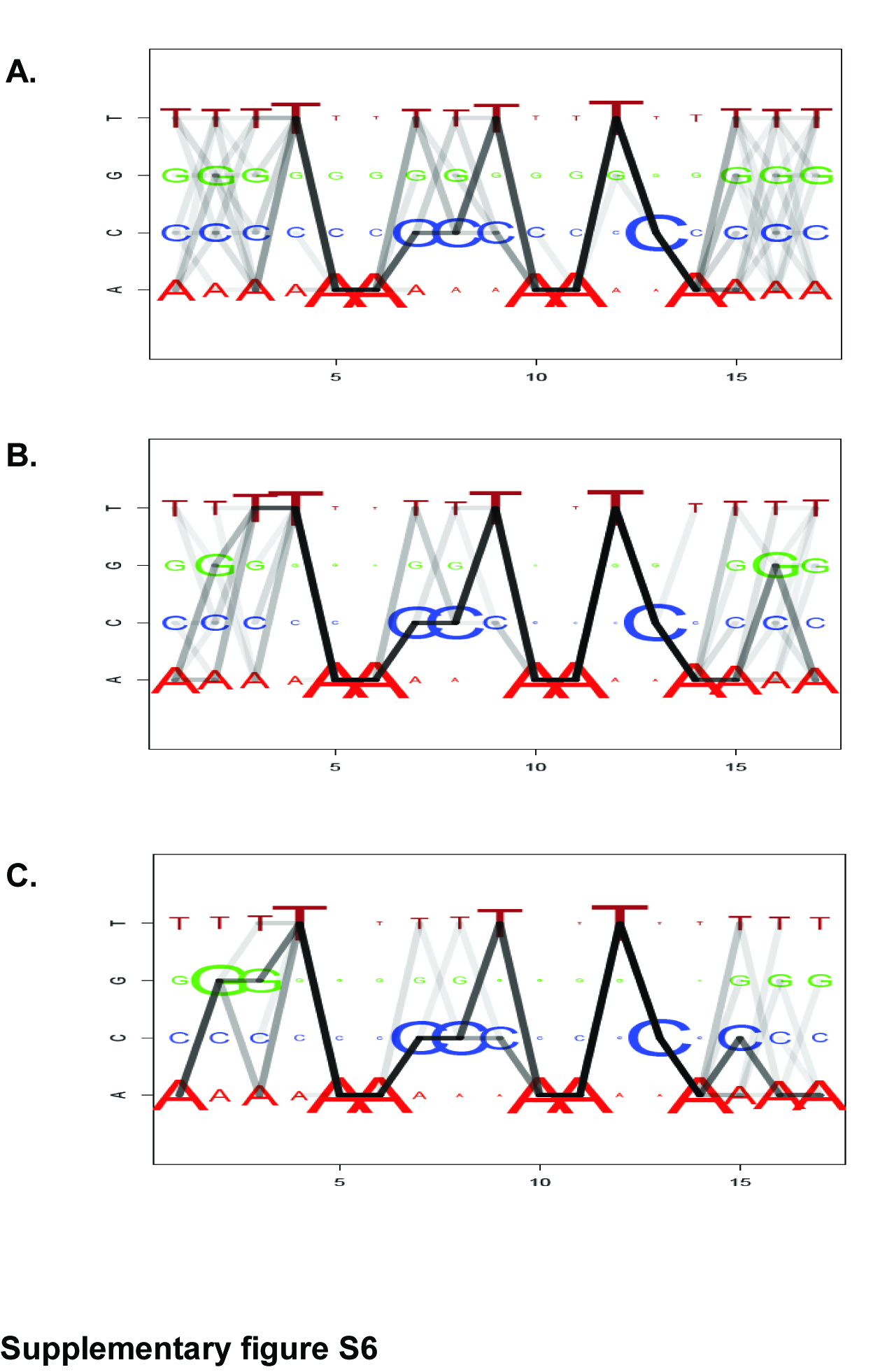

Supplement: Figure S6 — Consensus binding site sequence sequences of DUX4 at different genetic contexts. Position weight matrices of the DUX4 consensus binding sequence at A) unique binding sites, B) MaLR retrotransposons and C) L1 retrotransposons. The core double homeobox is conserved at all regions, flanking nucleotides show context specific variation. (TIF) [file pgen.1003415.s006.tif]

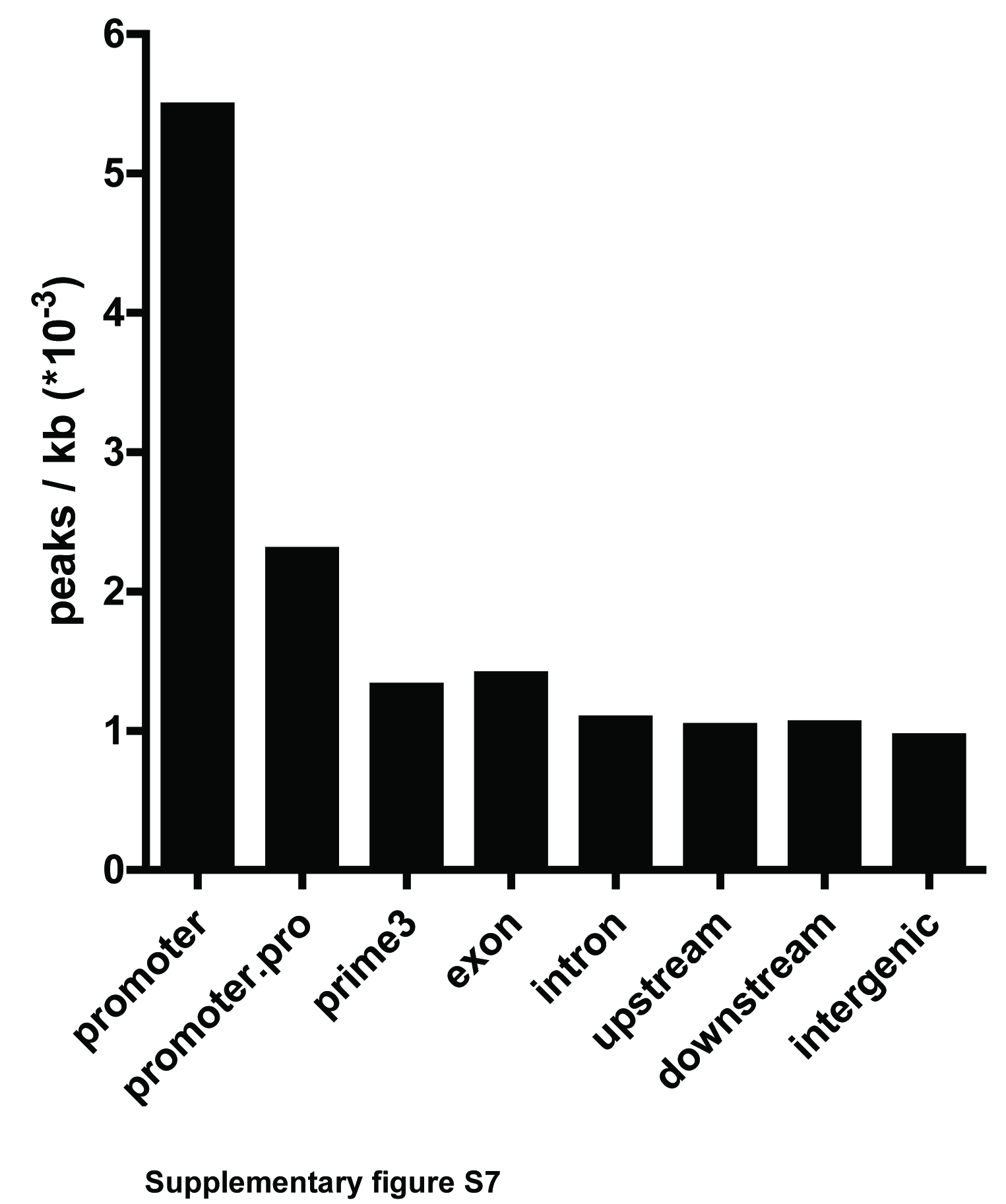

Supplement: Figure S7 — Distribution of DUX4 binding sites in the mouse genome. Relative distribution of ChIP peaks is displayed as the number of peaks per kb of total genomic sequence for each context. DUX4 shows a slight promoter bias as seen for transcription factors, but not for DUX4 in human myoblasts. (TIF) [file pgen.1003415.s007.tif]

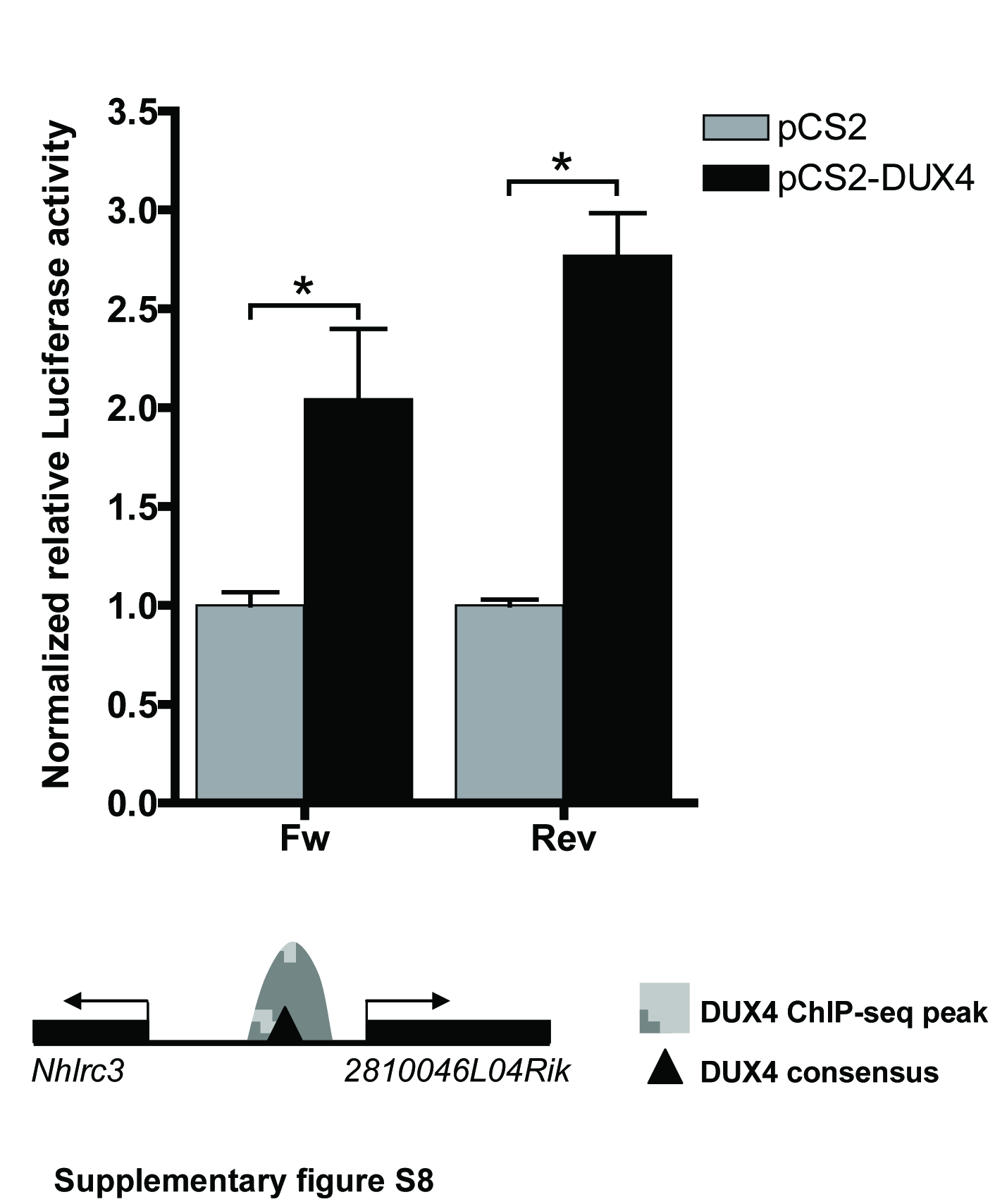

Supplement: Figure S8 — Luciferase reporter assays using a direct DUX4 target site. Normalized relative luciferase activity of the Nhlcr3 and 2810046L04Rik bidirectional DUX4 binding site in forward (Fw) and reverse (Rev) orientation in the absence (pCS2) or presence (pCS2-DUX4) of DUX4. Below a schematic overview of the DUX4 binding site is shown. All values are first normalized to pGL3 basic expression levels with or without DUX4. Next, the levels in DUX4 negative cells were set to 1. Error bars indicate SEM of quadruple measurements. Asterisks indicate p<0.05 according to a student's t-test. (TIF) [file pgen.1003415.s008.tif]

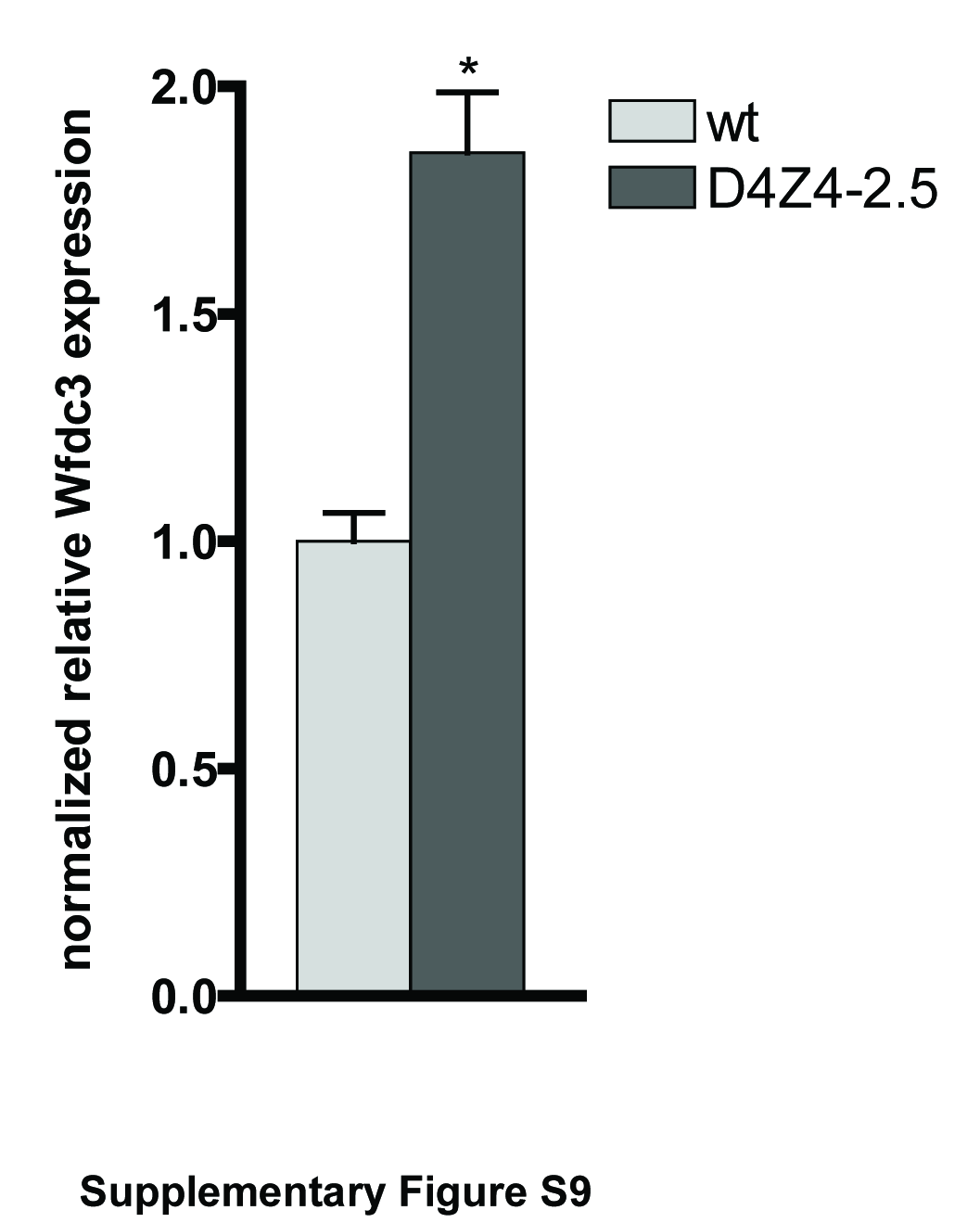

Supplement: Figure S9 — Expression analysis of the DUX4 target Wfdc3 in E9,5 embryos. Wfdc3 shows significant upregulation in D4Z4-2.5 embryos compared to wt controls (n = 6). Expression levels are normalized to the mouse reference gene Hprt and are plotted as the mean ± SEM. Asterisks indicate p<0.05 according to a student's t-test. (TIF) [file pgen.1003415.s009.tif]

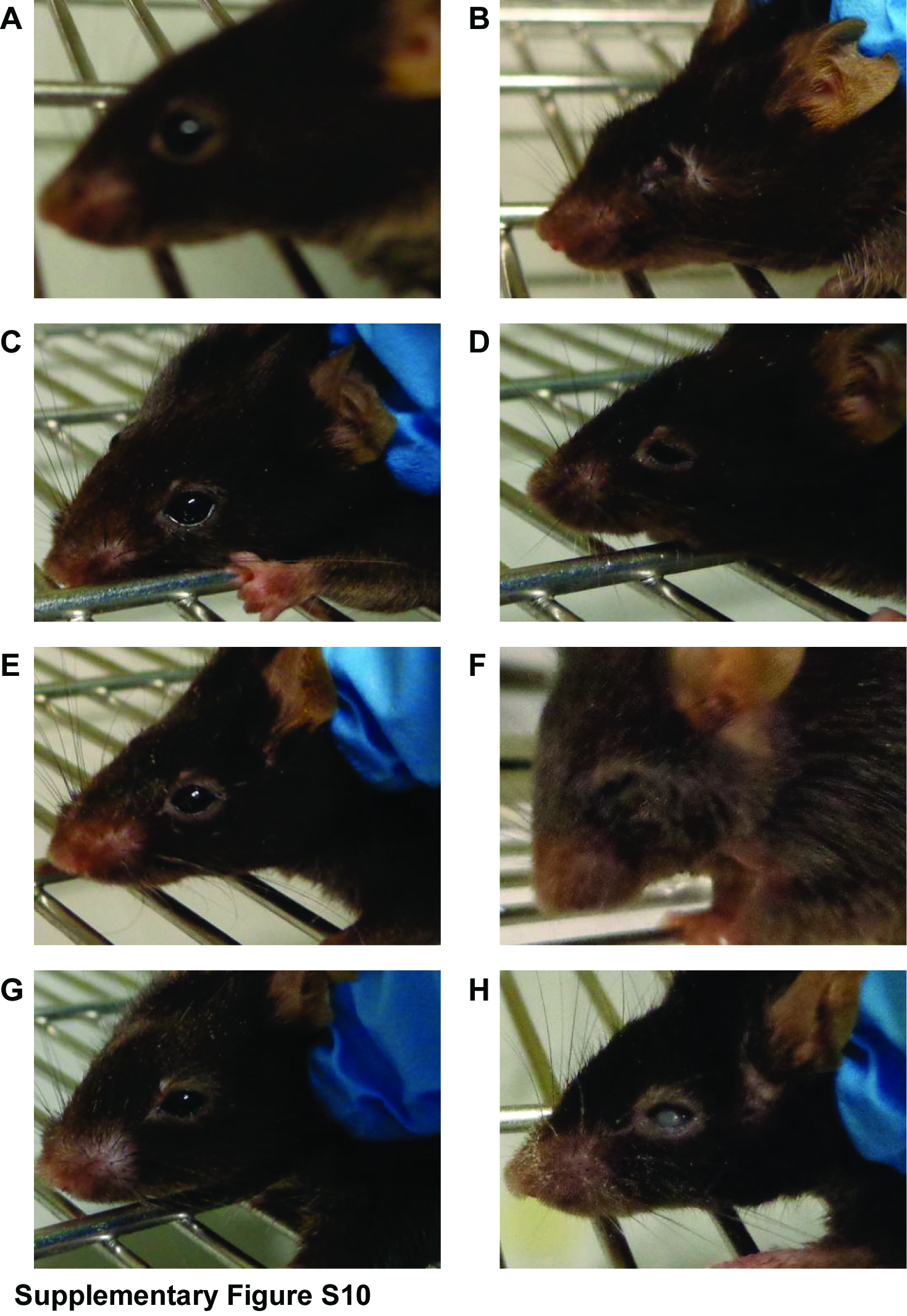

Supplement: Figure S10 — Keratitis in D4Z4-2.5 mice at different ages. Representative pictures of eyes of WT (A,C,E,G) and D4Z4-2.5 (B,D,F,H) mice at 11 weeks (A&B), 17 weeks (C&D), 27 weeks (E&F) and 1,5 years (G&H) of age. (TIF) [file pgen.1003415.s010.tif]
