# Supplementary material for: Intrinsic Epigenetic Regulation of the D4Z4 Macrosatellite Repeat in a Transgenic Mouse Model for FSHD
Source: PLoS Genet. 2013 Apr 4;9(4):e1003415. doi: 10.1371/journal.pgen.1003415 (PMC3616921; doi:10.1371/journal.pgen.1003415)
Supplement: Table S3 — Identified direct targets of DUX4 in C2C12 myoblasts. (PDF) [file pgen.1003415.s013.pdf]

Supplemental table S3. Identified direct targets of DUX4 in C2C12 mouse myoblasts

	ChIP-seq								*Expression*	
Chr.	start	end	max.cov	pval	bp from TSS	Entrez	Symbol	Strand	C2C12 logFC	C2C12 p-value fdr
1	65225085	65225360	11	5,54E-06	-412	15926	Idh1	+	0,974231746	2,51E-05
3	53267436	53267872	14	1,52E-07	-124	212127	2810046L04Rik	+	0,642507818	0,000241064
4	59819553	59819891	20	2,05E-10	1206	209131	Snx30	+	0,854824061	3,07E-05
4	151382229	151382550	13	5,13E-06	-602	242785	Klhl21	+	-0,894527959	4,91E-06
5	92512122	92512478	13	1,16E-06	-309	23881	G3bp2	+	1,154996839	0,000921265
5	143668205	143668532	13	7,15E-06	-27	11461	Actb	-	1,048563171	0,002115552
8	96910445	96910942	14	5,28E-07	378	64209	Herpud1	+	-0,909120547	1,08E-05
13	23666036	23666327	18	3,38E-10	121	319180	Hist1h2bf	-	0,650720299	4,89E-05
15	93425786	93426074	16	4,00E-08	-345	106042	Prickle1	-	2,180649772	6,37E-08
16	44116590	44116857	12	5,30E-07	-285	11964	Atp6v1a	-	0,891676629	1,68E-05
