# Supplementary material for: Intrinsic Epigenetic Regulation of the D4Z4 Macrosatellite Repeat in a Transgenic Mouse Model for FSHD
Source: PLoS Genet. 2013 Apr 4;9(4):e1003415. doi: 10.1371/journal.pgen.1003415 (PMC3616921; doi:10.1371/journal.pgen.1003415)
Supplement: Table S7 — List of primers and corresponding sequences. (PDF) [file pgen.1003415.s017.pdf]

Supplementary table S7: List of primers and corresponding sequences

MLPA probes

D4Z4 Fw	GGGTTCCCTAAGGGTTGGACTCCCGGAGTCCAGGATTCAGATCT
D4Z4 Rev	GGTTTCAGAATCGAAGGGCCAGGCATCTAGATTGGATCTTGCTGGCGC
FRG1 Fw	GGGTTCCCTAAGGGTTGGAGTACAAAACCTGGTTCACTGTTGCTGTGATATT
FRG1 Rev	TAAACTCACTGTTTGGAAGTCCAAAGGCATCTAGATTGGATCTTGCTGGCGC
p13E11 Fw	GGGTTCCCTAAGGGTTGGAGCCAGAGTTTGAATATACTGTGGTCATCTCTG
p13E11 Rev	CTCCAGTGCAAACTGTTTCCAGAATCTAGATTGGATCTTGCTGGCGC
FRG2 Fw	GGGTTCCCTAAGGGTTGGAGTTTGGATTGGGCTCCGATCCTGCTGCAAGAGAAGGT
FRG2 Rev	TTAGGTGACTCACCTCTCCCTAGGCAGAGTCTCTAGATTGGATCTTGCTGGCGC
Mm_CBP Fw	GGGTTCCCTAAGGGTTGGACCAGCTAGTGGAATTCAAAACACAATTGGTTCTGTTGGTGCA
Mm_CBP Rev	GGGCAACAGAATGCCACTTCCTTAAGTAACCCTCTAGATTGGATCTTGCTGGCGC
Mm_EP300 Fw	GGGTTCCCTAAGGGTTGGACCTAAGCACTGTTAGTCAGATTGACCCCAGCTCTAT
Mm_EP300 Rev	AGAGCGAGCTTACGCTGCTCTTGGACTACCCTATCATCTAGATTGGATCTTGCTGGCGC
Mm_EXT1 Fw	GGGTTCCCTAAGGGTTGGACAGCATGGCAAAGACTGGCAAAAGCACAAGGAT
Mm_EXT1 Rev	TCTCGCTGTGACAGAGACAACACCGAGTATGAGAAGTTCTAGATTGGATCTTGCTGGCGC
Mm_EXT2 Fw	GGGTTCCCTAAGGGTTGGACGAGAAACTGAAGAGCTTCCCCAACATTGGCAGCT
Mm_EXT2 Rev	TATGAAGCTTGCTGCACCTCCCGGGGCTTTCTAGATTGGATCTTGCTGGCGC

qPCR primers		Tm (˚C)
DUX4_fw	5’-CCCAGGTACCAGCAGACC -3’	62
DUX4_rev	5’-TCCAGGAGATGTAACTCTAATCCA-3’	
Hprt_fw	CGTCGTGATTAGCGATGATG	60
Hprt_rev	TTTTCCAAATCCTCGGCATA	
CyclophilinB_Fw	AAAAGGAAGACGACGGAGCC	60
CyclophilinB_Rev	TCGGAGCGCAATATGAAGGT	
Gapdh_Fw	TCCATGACAACTTTGGCATTG	60
Gapdh_rev	TCACGCCACAGCTTTCCA	
MyoG_fw	GCCCAGTGAATGCAACTC	60
MyoG_rev	GTCAGCCGCGAGCAAATG	
MyoD_fw	AACCCCAATGCGATTTATCA	58
MyoD_rev	CGAAAGGACAGTTGGGAAGA	
qPCR_D4Z4_fw (ChIP)	CCGCGTCCGTCCGTGAAA	65
qPCR_D4Z4_rev (ChIP)	TCCGTCGCCGTCCTCGTC	
Wfdc3_fw	CTTCCATGTCAGGAGCTGTG	60
Wfdc3_rev	ACCAGGATTCTGGGACATTG	
Zscan4c_fw	GATTATTGGCCACAGGACAAG	60
Zscan4c_rev	TCAGGGTGCTGTTCTTTCTG	
Ankrd1_fw	TCGACTCTTGATGACCTTCG	60
Ankrd1_rev	TTGCTTTGGTTCCACTCTGC	
Irf1_fw	AGCCGAAGACCTTATGAAGC	60
Irf1_rev	AGCAAGTATCCCTTGCCATC	
Nhlrc3_fw	AAGGACACTGGAGAGTGGTTAG	58
Nhlrc3_rev	GGGTGAACCTCACAGCAG	
2810046L04Rik_fw	CTCTTGGCCTCAAACATTG	58
2810046L04Rik_rev	AATAAATCTTCAATGGGACGAG	
Snx30_fw	ATTATGAGAAGTGCCTCATGG	58
Snx30_rev	GACATTCTGGTTCAGGGTTC	
Prickle1_fw	CTGCTGTCTGGAATGTGAG	60
Prickle2_rev	TGGTCCACACCAATATGC	
Med31_fw	GCTATGGAGACAGATGATGC	60
Med31_rev	AAATTAAGGTAGTTTGGGTTGG	
Gadd45g_fw	GCATCCTCATTTCGAATCC	60
Gadd45g_rev	CTCCTCGCAGAACAAACTG	
mdux_fw	GACCGAAGTCCAACTTGAGG	60
mdux_rev	CAGGTGTTGTGTCCATTTGC	
duxbl_fw	GCATCTCTGAGTCTCAAATTATGACTTG	60
duxbl_rev	GCGTTCTGCTCCTTCTAGCTTCT	
Psme4_fw	CAACTGGCAAGAACACAAGC	60
Psme4_rev	AATGAGCCCAGAATGAGGAC	
Trim36_fw	TGAAAGTGGGAGTTGCTTCC	60
Trim36_rev	GAATCAAAACAGGCGTCCTC	
Pvrl3_fw	TTGGCCAGACTGAACACTTG	60
Pvrl3_rev	TGCATTGTGCTCTCATCCTC	

Ccl7_fw	TCCCCAAGAGGAATCTCAAG	60
Ccl7_rev	CTTCCATGCCCTTCTTTGTC	
Id3_fw	ATCTCCCGATCCAGACAGC	60
Id3_rev	GTCAGTGGCAAAAGCTCCTC	
Line-1_fw	ACCCAAACCTATGGGACACA	60
Line-1_rev	CTGCCGTCTACTCCTCTTGG	
Orr1A_fw	AGCCCTTAGGAGGATTCCAA	60
Orr1A_rev	TGGTTCCACTCCCTGTTAGC	
Mte2b_fw	TGGGAAACTCACACCTTTCC	60
Mte2b_rev	CCTCATAGGCATGTGCAGAG	

cloning oligo's luciferase assay

Nhlrc3/2810046L04Rik DUX4 sense Fw	TCGAGACGCCCCAAACTAATCATTTGTGC
Nhlrc3/2810046L04Rik DUX4 sense Rev	GATCGCACAAATGATTAGTTTGGGGCGTC
Nhlrc3/2810046L04Rik DUX4 antisense Fw	GATCGACGCCCCAAACTAATCATTTGTGC
Nhlrc3/2810046L04Rik DUX4 antisense Rev	TCGAGCACAAATGATTAGTTTGGGGCGTC

**cloning primers Duxbl**		**Tm (˚C)**
BglII - **Duxbl** Fw	TCC**AGATCT**ATGGAGCTGAGCTGCAGTAC	58
SalI - **Duxbl** rev	CCG**GTCGAC**CTACGGAGTTTGGTGTGCTT	

**Genotyping transgenic mice**		**Tm (˚C)**
p13E11 fw	GAAAGCCCCCTGTGGGAG	55
p13E11 rev	TATAATTTTCTCTGGACTTCGC	
FRG1 fw	GCCATTGAAATGGATAAGCG	55
FRG1 rev	GGAATCAGATAATTTGACAGCC	

Haplotype sequencing

pLAM fw	AGCTCCTGGCGGTCAAAAGCA
pLAM rev	CAGGGGATATTGTGACATATCTCTGCACTCATC

Bisulphite primers

bis 1ste unit_fw	TAGGTAAAATTTTTTTAGAATTTTG
bis 1ste unit_rev	AAACAAATTAAAAAACCCCCTCTAC
bis upstream DUX4 ORF_fw	GTTTTTTTTGTTAGGTTTTAAAT
bis upstream DUX4 ORF_rev	CTTAAATATACCAAACCCTCTCTCC
