# Supplementary material for: Intrinsic Epigenetic Regulation of the D4Z4 Macrosatellite Repeat in a Transgenic Mouse Model for FSHD
Source: PLoS Genet. 2013 Apr 4;9(4):e1003415. doi: 10.1371/journal.pgen.1003415 (PMC3616921; doi:10.1371/journal.pgen.1003415)
Supplement: Text S1 — Supporting Materials and Methods and References. (PDF) [file pgen.1003415.s018.pdf]

Supporting text for:

Intrinsic epigenetic regulation of the D4Z4 macrosatellite repeat in a transgenic mouse model for FSHD

Yvonne D. Krom, Peter E. Thijssen, Janet M. Young, Bianca den Hamer, Judit Balog, Zizhen Yao, Lisa Maves, Laurie Snider, Paul Knopp, Peter S. Zammit, Tonnie Rijkers, Baziel G.M. van Engelen, George W. Padberg, Rune R. Frants, Rabi Tawil, Stephen J Tapscott, Silvère M. van der Maarel

Supporting Material & Methods

DUX4 RNA in situ hybridization on frozen tissue

To generate probes against either the 5' or 3' end of human *DUX4*, we cloned 574 base pairs (corresponding to nucleotides 6006-6579 of GenBank:FJ439133; [1]) of the 5' region of human *DUX4* (*DUX4-5'* probe) or 457 base pairs (corresponding to nucleotides 247-703 of GenBank:HQ266762 [2]) of the 3' region of *DUX4* (*DUX4-3'* probe) into TOPO 4.1 (Invitrogen). Plasmids were linearized and transcribed with a labeling kit (Roche) to make antisense digoxigenin-labeled RNA probes. 10-micron sections of OCT-embedded frozen testes tissue from control and D4Z4-2.5 mice were hybridized with antisense probes following standard procedures (http://goodrich.med.harvard.edu/resources/protocols/in_situ.pdf), with the modification that probe hybridization was carried out at 42°C.

Luciferase reporter assay

The binding sites at the direct target of DUX4 was cloned upstream of the firefly luciferase open reading frame in the pGL3 basic vector (Promega Benelux, Leiden, The Netherlands) using oligo annealing (for sequences: see supplementary table S7). Luciferase reporter assays were carried out in C2C12 mouse myoblasts. 30.000 cells per well were seeded in 12 well plates. 0.3 µg of the indicated reporter construct was co-transfected with 0.3 µg pCS2-DUX4 or 0.26 µg of the empty pCS2 vector to obtain equimolar transfection conditions. 24 hours after transfection cells were lysed in reporter lysis buffer and luciferase activity was assessed using the Dual Luciferase Reporter Assay system (Promega) according to manufacturer's instructions. Data is presented as firefly activity, corrected for the signal obtained by transfecting the empty pGL3 vector, and pCS2 signals were set to one. Data normalization by co-transfecting CMV-Renilla constructs was omitted because overexpression of DUX4 seemingly induced Renilla expression. Error bars represent SEM of a measurement in quadruple. Statistical significance was assessed by a two tailed independent sample student's t-test.

Histological examination

Mice predetermined for histopathology analysis were sacrificed at 9 months of age (3 WT and 3 D4Z4-2.5 mice). Full necropsy of the animals was performed. In particular also head muscles (Sternomastoid, masseter, temporalis and tongue muscles) were included. All tissue were immersed in 4% paraformaldehyde in phosphate buffered saline (0.1 M, pH 7.4) at room temperature for 24 hours, embedded in paraffin, and sectioned at 5 µm. Standard histological hematoxilin and eosin (HE) was performed.

Down-hill running induced muscle injury

WT and D4Z4-2.5 male mice, 9 weeks of age (n=9), each weighting 21-26g, were subjected to downhill (−15°) treadmill exercise using a motorized treadmill (Exert-6M-treadmill, Columbus instruments, Columbus OH, USA). Both WT and D4Z4-2.5 mice were acclimatized to the treadmill by

slowly increasing the speed (5 min at 10 meters per minute (mpm), then 5 min at 15 mpm, 5min at 20 mpm and 25 mpm) until fatigue. Fatigue was defined as the time when mice were no longer able or willing to keep up with the treadmill speed, despite continued gentle hand prodding for a period of 1 min. The treadmill running was performed 7 times during a 3 weeks time period, each day starting at 11 am. Mice were sacrificed by cervical dislocation 48 hrs after the last exercise and muscles were snap frozen in liquid nitrogen-cooled isopentane.

Evans Blue Dye uptake

Evans Blue dye (EBD) was prepared as a 1% wt/vol solution to yield 1 mg/g of body weight, was given via a separate intraperitoneal injection 7h before the start of the last exercise. The following day, mice were sacrificed and the muscles were quickly frozen in liquid nitrogen-cooled isopentane. Cryosections (10 µm thickness) were prepared from frozen triceps and gastrocnemius, fixed for 10 minutes in aceton. EBD positive fibers were revealed by fluorescence excitation at 615 nm.

Creatine kinase levels

Blood was collected from the tail vein in a minicollect tube (0.8 ml Lithium Heparin Sep, Greiner bio-one, Austria) through a small cut at the end of the tail, prior to exercise and 24 hrs after each exercise. The blood within the minicollect tubes was centrifuged for 5 minutes at 13.000 rpm at 4^0 C. CK levels were determined at same day of collection in a 1 to 10 times diluted plasma sample with Reflotron CK test strips in the Reflotron plus machine (Roche diagnostics Ltd., UK) according to manufacturer's instruction.

Forelimb grip strength test

The forelimb grip strength test was performed with a homemade grid attached to an isometric force transducer that measures peak force of the forelimbs (Ugo Basile, Italy). Mice were suspended above the grid, which they instinctively grasped, and then pulled backwards. Mice were tested five times, with three consecutive measurements per trial (15 in total), and a two minute interval between trials. The three highest measured values were averaged to calculate absolute strength, which was divided by the body weight in grams. Forelimb grip strength was assessed immediately prior and post exercise.

Two limb hanging wire test

Overall body coordination and strength was tested by a two limb hanging wire test. Mice were suspended above a metal cloth hanger secured 30 cm above a cage. The mouse was released a few seconds after instinctively grasping the wire with its forelimbs. Depending on the functional ability of the mouse, all limbs and the tail were used during a 10 min hanging session. Mice that fell down before the 10 min time limit, were given two more tries. The longest hanging time was used for analysis.

Cardiotoxin-Induced Muscle Injury

Mice were anesthetized with isoflurane. 50µl of a 10 µmol/L cardiotoxin solution (C3987; Sigma, St. Louis, MO) in phosphate-buffered saline (PBS) was injected into the gastrocnemius and triceps of female WT and D4Z4-2.5 mice using a 29G insulin needle. The contralateral muscle was injected with 50 µl of PBS to serve as control. The mice were sacrificed at 11 weeks of age by cervical dislocation at 10 (n=3) and 28 (n=3) days after cardiotoxin injection for histological analysis.

Assessment percentage fibrotic tissue

Gastrocnemius muscles of 11 weeks old WT and D4Z4-2.5 mice (n=3) were dissected and snap frozen in 2-methylbutane (Sigma–Aldrich, The Netherlands) cooled in liquid nitrogen 10 and 28 days after cardiotoxin injection, respectively. Sections of 8 µm were cut with a Shandon cryotome (Thermo Fisher Scientific Co., Pittsburgh, PA, USA) on Superfrost Plus slides (Thermo Fisher Scientific, Menzel-Gläser, Germany). Sections were fixed for five minutes with ice-cold acetone and stained with Harris haematoxylin and eosin (H&E) (Sigma–Aldrich, The Netherlands) according to conventional histological procedures. Sections were examined with a light microscope (LeicaDMLB, Leica Microsystems, The Netherlands) at a 2.5x magnification. Images were captured with a Leica DC500 camera and Leica IM50 software (Leica Microsystems, The Netherlands). Blending and background correction was performed with Adobe Photoshop CS3 version 10.0.1. Freely available ImageJ software with the haematoxylin/eosin (H&E) colour deconvolution plugin (Rasband, W.S., ImageJ, US National Institutes of Health, Bethesda, Maryland, USA, http://rsb.info.nih.gov/ij/, 1997–2008) was used to determine the fibrotic/necrotic percentage of the entire cross section as described previously [3]. This plugin provides a number of ‘‘built in” stain vectors to provide an accurate stain separation of the green, blue and red component, which is based on the primary and secondary three-dimensional color space (RGB/CYM) (http://www.dentistry.bham.ac.uk/landinig/software/cdeconv/cdeconv.html). Based on this linear description, ImageJ plugins have been created for different histological stainings [4]. The total tissue area was determined on the original picture while the area of healthy cells was determined using ImageJ’s automatic threshold on the eosin component, obtained after H&E colour deconvolution. This allowed calculation of the percentage of fibrosis/necrosis ([whole area - healthy area] / [whole area]).

Assessment fiber size and percentage centered nuclei

Fiber size and the proportion of centrally nucleated fibers were determined with Mayachitra Imago 1 (http://www.mayachitra.com/imago/) on whole muscle cross sections of the extensor digitorum longus (EDL) and gastrocnemius. Sections were stained with rabbit-anti-laminin (ab11575, 1:100, Abcam, USA), goat-anti-rabbit Alexa 594 (1:1000, Invitrogen, The Netherlands) and embedded in polymount with 500 ng/ml DAPI. Sections were examined and the middle section of the muscle was captured with a fluorescent microscope (Leica DM 5500B) and a Snapshot camera (Photometrics Coolsnap K4) at a 5 times magnification. Images were mosaicked with the Mayachitra Imago 1 (http://www.mayachitra.com/imago/) and preprocessed with ImageJ to obtain an even illumination of laminin. Pictures were segmented by the Mayachitra Imago software based on the staining intensity

difference between the membranes and the cytoplasm. Of these segmented fibers, the area (µm2), fiber density and the percentage of central nucleation were determined. Fibers were assessed for presence of centralized nuclei based on the maximum intensity (from the blue channel) of the cytoplasm. Objects smaller than 200 µm2 were excluded from analysis. Fiber density was determined by dividing the total number of fibers by the total area of the cross section.

Supplemental methods references

1. Snider L, Asawachaicharn A, Tyler AE, Geng LN, Petek LM, et al., (2009) RNA Transcripts, miRNA-sized Fragments, and Proteins Produced from D4Z4 Units: New Candidates for the Pathophysiology of Facioscapulohumeral Dystrophy. Hum Mol Genet 18: 2414-2430.

2. Snider L, Geng LN, Lemmers RJ, Kyba M, Ware CB, et al., (2010) Facioscapulohumeral dystrophy: incomplete suppression of a retrotransposed gene. PLoS Genet 6: e1001181. 10.1371/journal.pgen.1001181 [doi].

3. van PM, de WC, van Roon-Mom W, van Ommen GJ, 't Hoen PA, etal., (2010) A 3 months mild functional test regime does not affect disease parameters in young mdx mice. Neuromuscul Disord 20: 273-280.

4. Ruifrok AC, Katz RL, Johnston DA (2003) Comparison of quantification of histochemical staining by hue-saturation-intensity (HSI) transformation and color-deconvolution. Appl Immunohistochem Mol Morphol 11: 85-91.
